# Personal Electronic Records of Medications (PERMs) for medication reconciliation at care transitions: a rapid realist review

**DOI:** 10.1186/s12911-021-01659-8

**Published:** 2021-11-03

**Authors:** Catherine Waldron, Joan Cahill, Sam Cromie, Tim Delaney, Sean P. Kennelly, Joshua M. Pevnick, Tamasine Grimes

**Affiliations:** 1 School of Pharmacy and Pharmaceutical Sciences, Trinity College Dublin, Dublin, Ireland; 2 Centre for Innovative Human Systems & School of Psychology, Trinity College Dublin, Dublin, Ireland; 3 Pharmacy Department, Tallaght University Hospital, Dublin, Ireland; 4 Department of Medical Gerontology, Trinity College Dublin, Dublin, Ireland; 5 Cedars-Sinai Medical Center, Los Angeles, CA USA

**Keywords:** Medication reconciliation, Care transitions, Realist review, Electronic health records

## Abstract

**Background:**

Medication reconciliation (MedRec), a process to reduce medication error at care transitions, is labour- and resource-intensive and time-consuming. Use of Personal Electronic Records of Medications (PERMs) in health information systems to support MedRec have proven challenging. Relatively little is known about the design, use or implementation of PERMs at care transitions that impacts on MedRec in the ‘real world’. To respond to this gap in knowledge we undertook a rapid realist review (RRR). The aim was to develop theories to explain how, why, when, where and for whom PERMs are designed, implemented or used in practice at care transitions that impacts on MedRec.

**Methodology:**

We used realist methodology and undertook the RRR between August 2020 and February 2021. We collaborated with experts in the field to identify key themes. Articles were sourced from four databases (Pubmed, Embase, CINAHL Complete and OpenGrey) to contribute to the theory development. Quality assessment, screening and data extraction using NVivo was completed. Contexts, mechanisms and outcomes configurations were identified and synthesised. The experts considered these theories for relevance and practicality and suggested refinements.

**Results:**

Ten provisional theories were identified from 19 articles. Some theories relate to the design (T2 Inclusive design, T3 PERMs complement existing good processes, T7 Interoperability), some relate to the implementation (T5 Tailored training, T9 Positive impact of legislation or governance), some relate to use (T6 Support and on-demand training) and others relate iteratively to all stages of the process (T1 Engage stakeholders, T4 Build trust, T8 Resource investment, T10 Patients as users of PERMs).

**Conclusions:**

This RRR has allowed additional valuable data to be extracted from existing primary research, with minimal resources, that may impact positively on future developments in this area. The theories are interdependent to a greater or lesser extent; several or all of the theories may need to be in play to collectively impact on the design, implementation or use of PERMs for MedRec at care transitions. These theories should now be incorporated into an intervention and evaluated to further test their validity.

**Supplementary Information:**

The online version contains supplementary material available at 10.1186/s12911-021-01659-8.

## Introduction

Medication reconciliation (MedRec), a process to reduce medication error whenever patients transfer between care settings, is labour- and resource- intensive and time-consuming. MedRec has proven challenging to implement [1]. Lack of access to accurate information on patients’ medicine use increases the risk of medication errors. Medicine-related problems (MRP) such as side effects, inappropriate use and errors are a serious threat to patient safety. MRPs reduce quality of life, cause morbidity, death and increase health care costs [2–4]. There have been repeated calls for integration of health information systems including what we have termed Personal Electronic Records of Medications (PERMs) across sectors of care and between healthcare practitioners to facilitate MedRec [1, 5–8].

Whilst evidence is emerging regarding the positive impact of PERMs implemented in research environments, relatively little is known about the design, use or implementation of PERMs at care transitions that impacts on MedRec in the ‘real world’ [9, 10]. We aimed to respond to this gap in knowledge by undertaking a rapid realist review (RRR) of the literature. We used a conceptual framework developed by Burns [11] following his review of the literature of health information technology (HIT) systems which identified a number of themes that should be considered: Design, Implementation and Use [11].

We used the following definitions of the key terms: **Medication Reconciliation** (MedRec) relates to any opportunity taken to collect a medication history, check for any differences with current medication and communicate about any differences, thus creating a current accurate list; **Care Transition** relates to any movement between care settings or change in responsibility of care of a patient; **PERMs** relate to any digital record, partial or complete, of information regarding an individual’s medications (past or current) prescribed, dispensed or used by a patient; **Users** relates to any person, including healthcare workers, administrators or patients and carers, using PERMs.

## Methodology

Controlled trials generally determine what effect an intervention has when a number of static variables are applied but may not always identify how and why it worked [12]. In the complex world of healthcare service delivery, it is rare if the same intervention works in the same way in different contexts; it is important that these underlying causal pathways are considered to ensure that the intervention can be repeated with consistent outcomes. This is important in situations where an individual’s or organisation’s motivations and setting may influence the outcome.

Other researchers have examined the effectiveness of using PERMs to improve MedRec at care transition [13–15]. Realist methodology requires the researcher to use a different lens, in order to discover that which cannot be seen, allowing a deeper insight into the process of systematically and transparently synthesizing relevant literature in order to understand and develop theories (the unit of analysis in a realist review) about how and why things work or not, as well as what effect it has [16]. These "developed theories" are distinct from any existing theories, models or frameworks in the health informatics literature which were developed using alternative methods such as action research, for example the Clinical Adoption Meta-Model, or measurement scale validation, such as the Technology Acceptance Model. However, any such existing frameworks identified in the included studies were recorded and are considered in the discussion [17, 18].

A realist approach is suited to the synthesis of evidence about complex, multifaceted interventions because it explores how the underlying contexts and mechanisms configure to generate an outcome [19]. Realist methodology results in an explanation as opposed to a judgment about how interventions work [20]. More specifically, a realist review aims to identify what it is about interventions that generate change (i.e., the mechanisms) and under which circumstances the mechanisms are triggered (i.e., the contexts), which result in changes in the behaviour of the participants and/or implementers of the intervention (i.e., the outcome). It aims to explore an intervention’s intended and unintended outcomes and to explain successes, failures and partial successes. These three elements, context, mechanism and outcome, are presented together as a statement or theory which attempts to describe what needs to happen for the intervention to work, i.e. a Context Mechanism Outcome Configuration (CMOC) [16]. The products of realist reviews are theories, often produced in the form of “if …. then” statements developed from one or more CMOCs found in the available evidence.

A RRR is a more focused and accelerated version of a full realist review which aims to produce theories in a time-sensitive way and that is useful to a specific audience about emerging issues, while preserving the core elements of realist methodology [21].

Interventions are influenced by an endless source of contexts which can, for convenience, be grouped under the four I’s as outlined by Pawson et al. [22]; (1) Individuals—the characteristics and capacities of the various stakeholders in the intervention; (2) Interpersonal relations—the stakeholder relationships that carry the intervention; (3) Institutional settings—the rules, norms and customs local to the intervention; (4) Infrastructure—the wider social, economic and cultural setting of the intervention.

For this RRR, ***contexts*** represent conditions and examples include, but are not limited to, issues such as work environment, resources (i.e. investment, equipment, staffing, training), governance, policies and standards, interoperability, sources of information, accuracy, reliability, security, user interface, user access, user (computer) skills and frequency of use of PERMs, user workload and readiness to change, patient consent, implementation, evaluation and audit.

In this RRR, ***mechanisms*** are about individuals’ or organisations’ beliefs / feelings about PERMs and related contexts (as listed above). Examples of positive mechanisms include, but are not limited to; being enabled, engaged, involved, trusting, satisfied, contented, valued, proud, determined, confident, supportive, ready, motivated, aware, skilled, incentivised or efficient. A similar range of negative mechanisms may also be at play.

The ***outcomes*** for this RRR will include anything that has impacted positively or negatively on the use of PERMs for medication reconciliation at care transitions. Examples of outcome topics might include but are not limited to: workflow, communication, frequency of use, relationship between stakeholders (patients, pharmacists, GPs, hospital staff), efficiency, errors, adherence to medications, patient’s awareness of medications and reasons for use.

The approach is guided by methodological guidance, publication standards and training materials for realist and meta-narrative reviews: Realist And Meta-narrative Evidence Syntheses: Evolving Standards (RAMESES), which have been followed in this review [23].

### Methods

This rapid realist review was undertaken over a seven-month period from August 2020 to February 2021. The protocol for the RRR was registered with Prospero in September 2020 (Additional file 1). This project was funded by The Meath Foundation Research Grant 2019.

Keeping in mind that the steps in a RRR are iterative and there may be reason to look back and revise steps already undertaken as the data from the literature is revealed, the following steps were undertaken.

### Formation of a reference panel and expert panel

A vital part of the RRR is partnering with people ‘on the ground’, providing local knowledge and context (the Reference Panel), and experts in the field from around the world (the Expert Panel) who ensure we reflect the most current thinking on the topic. For this RRR the Reference Panel was made up of thirteen key stakeholders, providing insight from clinicians, safety science, informatics, human factors expertise, e-health, governance, policy, research and academia. The Expert Panel comprised five key researchers in the area, from the USA, Ireland, Sweden and UK.

At the beginning of the RRR both panels were asked to provide key articles on the topics of interest. The Reference Panel was also asked to collaborate in identifying the challenges and facilitators for introducing PERMs for MedRec at care transition. The information provided was reviewed for emerging themes, which assisted the research team to develop the research questions, which sought to identify the types of medication data sources used, the contexts and mechanisms that impact on the outcomes relating to the design, implementation and use of PERMs for MedRec, with the intention of identifying in what circumstances the use of PERMs for MedRec in care transitions are most likely to be effective.

### Search strategy and study selection

Firstly, we used search terms based on those used for a systematic review of MedRec at care transitions completed by a member of this research team in 2018 [24] and we supplemented these with additional terms for care transition/ care continuity, medication errors and human/computer interaction (Additional file 2). We limited the searches to articles in the English language, articles were excluded if no abstract was available. The databases searched were Pubmed, Embase, CINAHL Complete and OpenGrey. The searches included any articles identified up to the 1^st^ of September 2020.

Purposeful searching, particularly for qualitative reports, interviews or surveys and reports of negative findings continued throughout the review. We also considered relevant articles suggested by the Reference and Expert Panels. All article types were eligible for inclusion, for example, policy documents, newspaper articles and opinion pieces, no study designs were excluded. The reference lists of relevant articles were considered (chaining) and snowballing was also carried out to a small extent. The protocol outlined that relevant review articles would be searched for relevant articles not already included, if numbers are low, this however was not required.

We piloted the screening of title and abstracts, which resulted in some amendments and clarifications of the inclusion and exclusion criteria (Additional file 3). The remaining titles and abstracts were screened independently by at least two of the four screeners. A sample (10%) of the full text articles were then screened by two reviewers to ensure consistency, and disagreements were resolved by a 3rd reviewer. The remaining articles were screened by one screener, an acceptable process in a RRR, using the Covidence software package [25]. We used the NVivo software programme to manage the data extraction from the included full texts articles.

### Quality assessment

Quality assessment (QA) of realist data is considered under the headings of relevance, rigour and richness [20, 26]. Richness was scored for those articles that met all inclusion criteria at the full text stage (N = 94). Relevance and Rigour was scored for those articles included in the review (N = 51). A sub-sample of the articles included for full text review (10%) was quality assessed by two reviewers independently and any disagreements discussed and resolved. The QA process to be applied by one reviewer to the remaining articles was refined.

Relevance was assessed by determining if the article had information of value to the review. Rigour was assessed based on whether the sources or methods used to generate the relevant data were credible and trustworthy. We scored the relevance and rigour of the included articles using the following ratings: 0 = very poor, 1 = poor, 2 = good, 3 = very good.

Richness, a term coined by Booth et al., relates to the level of theoretical and conceptual development detail provided in the articles, and used as a means to identify articles of most value in a realist review. We assessed it by scoring the articles in relation to the richness relative to the research questions. To score highly an article should provide sufficient details in relation to how the approach used was expected to work; documenting the process and explaining contextual factors that influenced implementation and/or outcomes [26]. We rated the richness as follows: 0 = nothing of interest, not focused on design, implementation or use, 1 = limited data of interest, likely to appear in other articles, 2 = limited data of interest, but quick to extract it and could add weight to findings, 3 = some good quality data, 4 = Much valuable data. The richness assessment at full text reading allowed us to identify the articles with the most potential for providing rich data. This was the method we used which ultimately decided which of the articles were included in this rapid realist review, meeting the implicit time limitations. The richness rating was revised at the data extraction stage, some articles had their richness rating revised downwards during this process (Additional file 10).

### Extraction of the data

The data extraction process took place between September and December 2020 following a brainstorming session to finalise the list of themes identified by the conceptual HIT systems framework developed by Burns [11], the reference panel suggestions and some additional themes identified in the literature (Additional file 4) [11]. This allowed us to make decisions on how the data would be managed in NVivo using broad headings or *Nodes* based on the themes.

A pilot extraction of data from three articles was undertaken in stages by three team members independently. After the data extraction was completed for the first article, we discussed the findings and how the NVivo set up was working. We then completed the data extraction independently for the two remaining articles and made final refinements to the process. The data from the remaining 16 articles was extracted by one team member. The NVivo nodes developed iteratively over the extraction process, the final NVivo codebook used is in Additional file 5.

We also extracted additional data—including the authors, year and country the study was carried out, the research questions or objectives, the formal outcomes, study conclusions, any theory/concept outlined or inferred, the software in use, the setting (hospital, nursing home etc.) and personnel involved and any citations of interest. We reassessed the quality assessment ratings during the extraction process and recorded them. This was based on individual sections of extracted data rather than the overall article, as per RAMESES guidelines [23]. We used an agreed template to produce a summary document, including this additional information, for each article (Additional file 6).

### Analysis of the data

We analysed the extracted data to find and align evidence to demonstrate that particular mechanisms influence particular outcomes in particular contexts, i.e. CMOCs which form the basis of provisional theories [27] (Additional file 7).

The analytical processes used to make sense of the CMOCs being developed followed a process outlined by Pawson [28]:

- Juxtaposing—where evidence about mechanisms in one source enables insights into outcome patterns of another source.
- Reconciling—finding explanations for different outcomes by uncovering contextual differences.
- Adjudication—explaining opposing study outcomes on the basis of methodological strengths and weaknesses.
- Consolidation—where outcomes differ in particular contexts and explanations can be constructed of how and why these differences occurred.
- Situating—describing which mechanisms were activated in which context.

The result is a series of theories based on the literature examined which describe what is it about how, why, when, where and for whom PERMS are designed, used or implemented in practice at care transitions that impacts on medical reconciliation.

A survey to determine the reference panel’s feedback on the provisional theories was piloted and revised. The expert and reference panels were then provided with a brief introduction to realist methodology and the purpose of the RRR (Additional file 8) and asked to complete the survey (Additional file 9). They were asked to rate the theories on a scale of 1 -5 (1 = lowest, 5 = highest) in relation to: how well they **understood** each theory, the **relevance** of the theory and the **feasibility** to apply the theory in practice. Comments or suggestions to improve theory clarity and focus were invited.

## Results

### Description of dataset

**Fig. 1:**
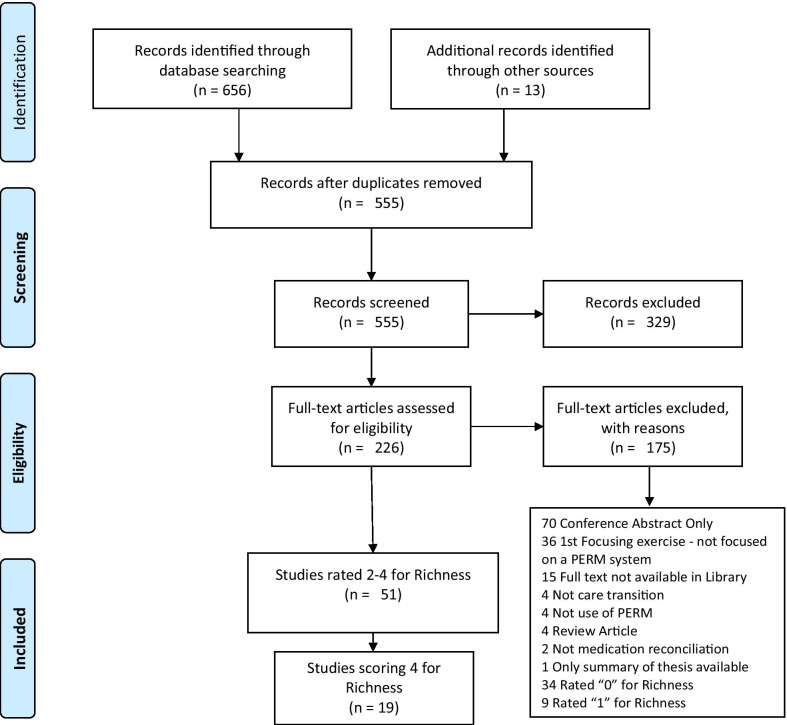
Included articles/ prisma flow chart

The final review included nineteen articles (Fig. 1) with a richness score of 4 assessed at full text reading stage. This score ensured that the included articles contained a high level of theoretical and conceptual development detail allowing refinement of the theories. All but two of the included articles rated good or very good for both relevance and rigour (Table 1).

**Table 1:** Provisional and final theories with summary synthesis

Provisional Theory	Articles contributing to theory	Data synthesis summary	Panel Ratings	Final Theory, text in BOLD highlights changes	Relevance of Theory to Stages of PERM
**1. Engage Stakeholders**					
If users are given the opportunity to provide input, and both give and receive feedback at all stages of the introduction of a PERM system, they will feel engaged, be supportive and understand the challenges, they will then accept and feel confident about using a PERM system to complete MedRec at care transitions	1, 2, 3, 4, 5, 9, 10,13, 14, 15, 16, 17, 18, 19	There was a general consensus from the articles that engagement of the stakeholders was important. Elements such as the level of engagement, opportunities to provide feedback in a meaningful way, a bottom up/top down approach allowing cross department discussions, as well as the equal consideration of technology and clinical elements, helped to describe what this engagement should look like. Setting up innovative ways for stakeholders to give feedback throughout the process and showing them how the feedback was used were identified as ways to achieve this engagement	**U: 4.5** **R: 4.4** **P: 3.8**	If **stakeholders including all user groups** are given the opportunity to provide input, and both give and receive feedback at all stages of the **design, implementation and use of** PERMs, they will feel engaged, be supportive and understand the challenges, they will then accept and feel confident about using PERMs to complete MedRec at care transitions	Design, implementation and use
**2. Inclusive design**					
If a PERM system is developed using user-centered design & usability principles then users will feel heard and supported, thus fostering successful collaboration, acceptance and increased use of the PERM system to complete MedRec at care transitions	1, 2, 4, 6, 7, 8, 9, 11,15, 16, 17, 18, 19	The display features, layout and use of abbreviations, and terminology or drug databases were the main topics discussed in the literature in relation to the importance of involving others in collaborative design. Issues such as phasing the introduction of PERMs, considering the electronic systems already in use and the early development of solutions to allow complex systems cater for the needs of multiple users and functions, were all considered to impact on the successful implementation and use of PERMs	**U: 4.5** **R: 4.5** **P: 4.2**	If PERMs are designed **with user input** and employing user-centered design & usability principles then users will feel heard and supported, thus fostering successful collaboration, acceptance and increased use of PERMs to complete MedRec at care transitions	Design
**3. PERMs complement existing good processes**					
If the content of a PERM system replicates MedRec processes and forms that are already in existence in a setting and have been shown to work well, then the PERM will feel familiar and consistent, users will feel confident using it and the PERM will become embedded more easily into normal work practices, allowing a smooth transition to PERM to improve MedRec at care transitions	4, 6, 8, 12, 13, 14, 15, 18, 19	The importance of firstly ensuring that the systems being used for Med Rec are fit for purpose before any plans to design the PERMs was stressed in a number of the articles. This includes identifying who is responsible for at each stage of the MedRec process	**U: 4.4** **R: 3.9** **P: 3.5**	If PERMs **complement** MedRec **cognitive and workflow** processes **or** forms that are already in existence in a setting and have been shown to work well, then PERMs will feel familiar and consistent, users will feel confident using it and PERMs will become embedded more easily into normal work practices, allowing a smooth transition to PERMs to improve MedRec at care transitions	Design
**4. Build Trust**					
If users are made aware of how others access and use the information on a PERM system, the integrity of the sources of data that populate the PERM and the integrity of how data are protected, their trust and confidence in the system will increase, they will then comprehend how the system aims to work and be more likely to use it at care transitions to improve MedRec and patient safety	1, 2, 3, 4, 5, 6, 8, 12, 13, 14, 15, 18, 19	Helping the user to understand the processes or roles of others, the sources, accuracy and safety of the medication data and the reliability of PERMs were shown in the data to impact on the level of trust in PERMs This theory was rated joint lowest for understanding by the panels. Of the panellists who provided some comment in relation to their ratings, some did not understand it and others understood it but did not agree with it, while others agreed with it completely	**U: 4.1** **R: 4.3** **P: 3.9**	If users are aware **and understand** how **they and** others access and use PERMs, the integrity of the PERMs data sources and the data protection **controls**, their trust and confidence in the PERMs **design and use** will increase, they will then be more likely to **value and** use it at care transitions to improve MedRec and patient safety	Design, Implementation and Use
**5. Tailored Training**					
If training is provided to users that takes into account their existing MedRec knowledge and skills, their computer skills and their role at care transitions, and the training outlines the clear benefits, usefulness and usability of a PERM system, users will then feel less anxious and be more engaged and confident in relation to the introduction of a PERM system in their setting	1, 2, 3, 4, 5, 6, 7, 8, 9, 10, 11, 12, 13, 14, 15, 16,17, 19	There was a general consensus regarding the need for training on any new PERMs. How this training should be tailored to different users was considered in some of the included articles. Making users aware of or reminding them about the impact of poor Med Rec at care transitions and taking into consideration their computer skills were common elements. These knowledge and skills should not be assumed	**U: 4.4** **R: 3.8** **P: 3.6**	No changes made	Implementation
**6. Support and on-demand training**					
If training on a PERM system is provided at implementation and continued at regular intervals to cater for new staff or those needing additional support, at times or in formats that suit all users, with the opportunity for users to give feedback and they are given time to become familiar with the system, then the users will feel supported and enabled to use the PERM system consistently thereby improving MedRec at care transitions	1, 4, 10, 12, 13, 14, 15, 16, 18, 19	A number of articles highlighted the need for continual or repeat training, catering for the individual needs of staff, to ensure that the standard of Med Rec using PERMs remained consistent. The use of dedicated staff, “super-users” or “champions”, freed from their normal duties, to provide this training and support was also identified. The opportunity for users to provide feedback in relation to the training offered to them was also considered important	**U: 4.4** **R: 3.6** **P: 3.4**	If **support and** training on PERMs is **available on demand** to cater for new staff or those needing additional support, at times or in formats that suit all users, with the opportunity for users to give feedback **on the training**, then the users will feel supported and enabled to use PERMs consistently thereby improving MedRec at care transitions	Use
**7. Interoperability**					
If the PERM data sources are technically interoperable with the system, allowing integration of data from multiple sources then users will find the system aligns with or improves the MedRec process flow, thereby increasing their use of PERM for MedRec at care transitions impacting positively on patient safety	1, 3, 4, 6, 7, 10, 11, 12, 16, 19	Interoperability was assumed in many of the articles, will little reference to it other than to define it and to acknowledge that the ability to integrate data from multiple sources was important in the usability of PERMs. PERMs must be seen to improve and support the safety and efficiency of MedRec at care transitions	**U: 4.6** **R: 4.8** **P: 4.0**	No changes made	Design
**8. Resource investment**					
If the required time and resource intensity of introducing and maintaining a PERM system for MedRec at care transitions is recognised and understood early by organisations and they acknowledge from the outset that it will increase the amount of data gathered, recorded and used, increasing the users’ workload, then organisations will be prepared and budget for the additional resources required	1, 3, 7, 8, 11, 12, 13, 15, 18	Some electronic resources have been shown to save money in the long run, for example e-prescriptions, this is not the case with MedRec. As reported in a number of the articles, improvement comes with a price; improving MedRec at care transitions increases the effort, volume and quality of data gathered which provides new opportunities for risk identification, management and analysis, resulting in the need for additional staff to carry out all of these elements and the budget to pay for them	**U: 4.1** **R: 4.2** **P: 2.9**	If the **increased effort, volume and quality of data gathered when using** PERMs for MedRec at care transitions, **providing opportunities for risk identification, management and analysis,** is recognised by **leaders/ management** from the outset then **they will understand the need for additional resourcing to support the use of PERMs to improve MedRec at care transitions and patient safety**	Design, Implementation and Use
**9. Positive impact of Legislation or Governance**					
If the introduction of a PERM system or standards for the MedRec process is supported by relevant legislation, governance or policies then organisational participation and engagement is increased impacting positively on individual users' engagement with the introduction of a PERM system to improve MedRec at care transitions	1, 2, 3, 4, 6, 7, 8, 10, 11, 12, 13, 16, 18, 19	The existence of legislation or governance was seen mainly as a positive influence in the articles. However, if the legislation or governance was slow to adapt to changes, for example in the expanding role of healthcare workers in the MedRec process, it could have a negative influence	**U: 4.5** **R: 4.2** **P: 3.7**	No changes made	Implementation
**10. Patients as users of a PERM**					
If patients are supported to use PERM to understand and record their medication use and share their medication information, they will feel enabled, empowered and organised in helping to maintain an accurate medication record, be more informed and have improved likelihood of adherence to their medications	1, 2, 6, 7, 8, 9, 11, 12, 13, 17, 19	There were conflicting opinions in relation to the role patients could play in Med Rec using PERM, with some authors in full support of their role while others had concerns. The main issues identified by healthcare workers were the accuracy of data supplied by patients and their ability to use technology. There were a small number of articles included in the review that dealt with this issue from the patient’s perspective. For the most part, patients were positive about the use of PERM for their health information, reporting increased understanding about their medications and improved adherence	**U:4.6** **P: 4.6** **P: 3.9**	No changes made	Design, implementation and Use

Articles contributing to theory: Numbers relate to articles as per Additional file 10

Panel Ratings: U: Understanding, R: Relevance, P: Practicality

The nineteen articles (Additional file 10) were published between 2006 and 2020. Seven articles covered all three elements of interest to this review; design, implementation and use of PERMs [29–35]. Four articles considered only design [36–39], four considered only implementation [40–43] and the remaining four considered only use [44–47].

Eight articles reported on the use of PERMS in the USA, three in the UK, two each in Australia and Austria, and one each in Belgium, Canada, Denmark and Sweden. The Austrian articles [40, 45] and two of the American articles [43, 46] were each reporting on different elements of the same intervention.

The articles reported on the use of local PERMs in a hospital or clinic setting (n = 7) or national PERMs (n = 8). Of the remaining four articles, two reported on patients use of PERMS, one on an electronic discharge system and one on the use of an e-messaging system.

The users of the PERMs were predominately hospital-based staff which included doctors, nurses, hospital pharmacists and technicians, administrators and IT staff. Of the fifteen articles involving hospital-based staff, seven involved hospital staff only. The other eight also involved GPs (n = 4), community pharmacists (n = 3), nursing home staff (n = 2), system designers (n = 1) and patients (n = 4). The remaining four articles involved a mixture of users who were patients and/or their families/carers (n = 3), GPs (n = 2), community pharmacists, (n = 1), or nursing home nurses (n = 1).

The location of PERMs use was influenced by who the users were, and so was mainly in hospitals only (n = 9), jointly in GP practices and community pharmacies (n = 4), jointly in hospitals and nursing homes (n = 2), jointly in hospitals and GP practices (n = 1) or jointly in all of these settings (n = 1). Use of PERMs by patients in their own home was reported in two articles.

### Data synthesis

The identified CMOCs from the 19 articles, allowed us to develop ten theories in relation to what is it about how, why, when, where and for whom PERMs are designed, implemented or used in practice at care transitions that impacts on medical reconciliation.

**Fig. 2:**
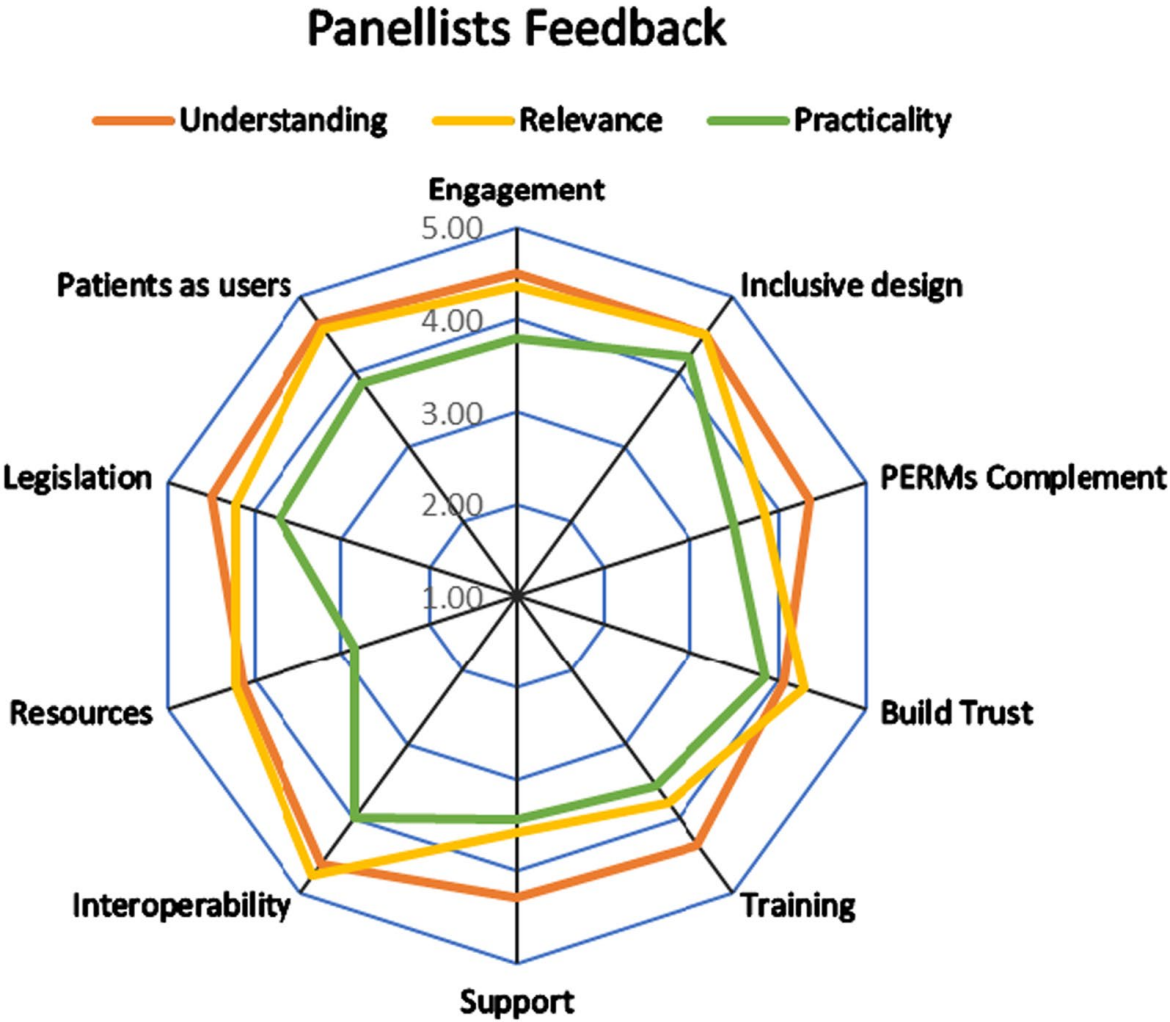
Panellists feedback

The panels’ feedback (88% response rate) (Fig. 2, Additional file 11) indicated that all of the theories were understood (average score 4.4, range 2–5). The panellists rated the theories an average 4.21 (range 2 to 5) for relevance and an average 3.68 (range 1 to 5) for practicality.

Panellists’ feedback comments related to clarifying that no one theory was the solution, the need for a good MedRec process should precede the introduction of PERMs to support it and highlighting the importance of evaluating the implementation and use of PERMs so that unintended effects and problems could be identified to improve the PERMs. Several panellists commented positively on the methodology used, their satisfaction with the range of theories developed and the ease at which they were able to provide their feedback. The panellists’ engagement facilitated our revision of the theories improving their clarity and intention: seven theories were revised and the remaining three required no change. None of the theories were considered irrelevant or totally impractical, although the challenge of implementing some of them was acknowledged.

The ten provisional and final theories and a data synthesis summary for each theory is presented in Table 1. The more detailed synthesis with direct extracts from the literature is provided in Additional file 12. The ten final theories are as follows:

1. **Engage stakeholders**—If stakeholders including all user groups are given the opportunity to provide input, and both give and receive feedback at all stages of the design, implementation and use of PERMs, they will feel engaged, be supportive and understand the challenges, they will then accept and feel confident about using PERMs to complete MedRec at care transitions.
2. **Inclusive design**—If PERMs are designed with user input and employing user-centered design & usability principles then users will feel heard and supported, thus fostering successful collaboration, acceptance and increased use of PERMs to complete MedRec at care transitions.
3. **PERMs complement existing good processes**—If PERMs complement MedRec cognitive and workflow processes or forms that are already in existence in a setting and have been shown to work well, then PERMs will feel familiar and consistent, users will feel confident using them and PERMs will become embedded more easily into normal work practices, allowing a smooth transition to PERMs to improve MedRec at care transitions.
4. **Build trust**—If users are aware and understand how they and others access and use PERMs, the integrity of the PERMs data sources and the data protection controls, their trust and confidence in the PERMs design and use will increase, they will then be more likely to value and use it at care transitions to improve MedRec and patient safety.
5. **Tailored training**—If training is provided to users that takes into account their existing MedRec knowledge and skills, their computer skills and their role at care transitions, and the training outlines the clear benefits, usefulness and usability of PERMs, users will then feel less anxious and be more engaged and confident in relation to the introduction of PERMs in their setting.
6. **Support and on-demand training**—If support and training on PERMs is available on demand to cater for new staff or those needing additional support, at times or in formats that suit all users, with the opportunity for users to give feedback on the training, then the users will feel supported and enabled to use PERMs consistently thereby improving MedRec at care transitions.
7. **Interoperability**—If the data sources are technically interoperable with PERMs, allowing integration of data from multiple sources, then users will find that PERMs align with or improve the MedRec process flow, thereby increasing their use of PERMs for MedRec at care transitions impacting positively on patient safety.
8. **Resource investment**—If the increased effort, volume and quality of data gathered when using PERMs for MedRec at care transitions, providing opportunities for risk identification, management and analysis, is recognised by leaders/ management from the outset then they will understand the need for additional resourcing to support the use of PERMs to improve MedRec at care transitions and patient safety.
9. **Positive impact of legislation or governance**—If the introduction of PERMs or standards for the MedRec process are supported by relevant legislation, governance or policies then organisational participation and engagement is increased impacting positively on individual users' engagement with the introduction of PERMs to improve MedRec at care transitions.
10. **Patients as users of PERMs**—If patients are supported to use PERMs to understand and record their medication use and share their medication information, they will feel enabled, empowered and organised in helping to maintain an accurate medication record, be more informed and have improved likelihood of adherence to their medications.

The theories are all interdependent to a greater or lesser extent. It is likely that several or all of the CMOCs inherent in the theories would need to be in play in order for them to collectively impact on the design, implementation or use of PERMs for MedRec at care transitions in a positive way.

**Fig. 3:**
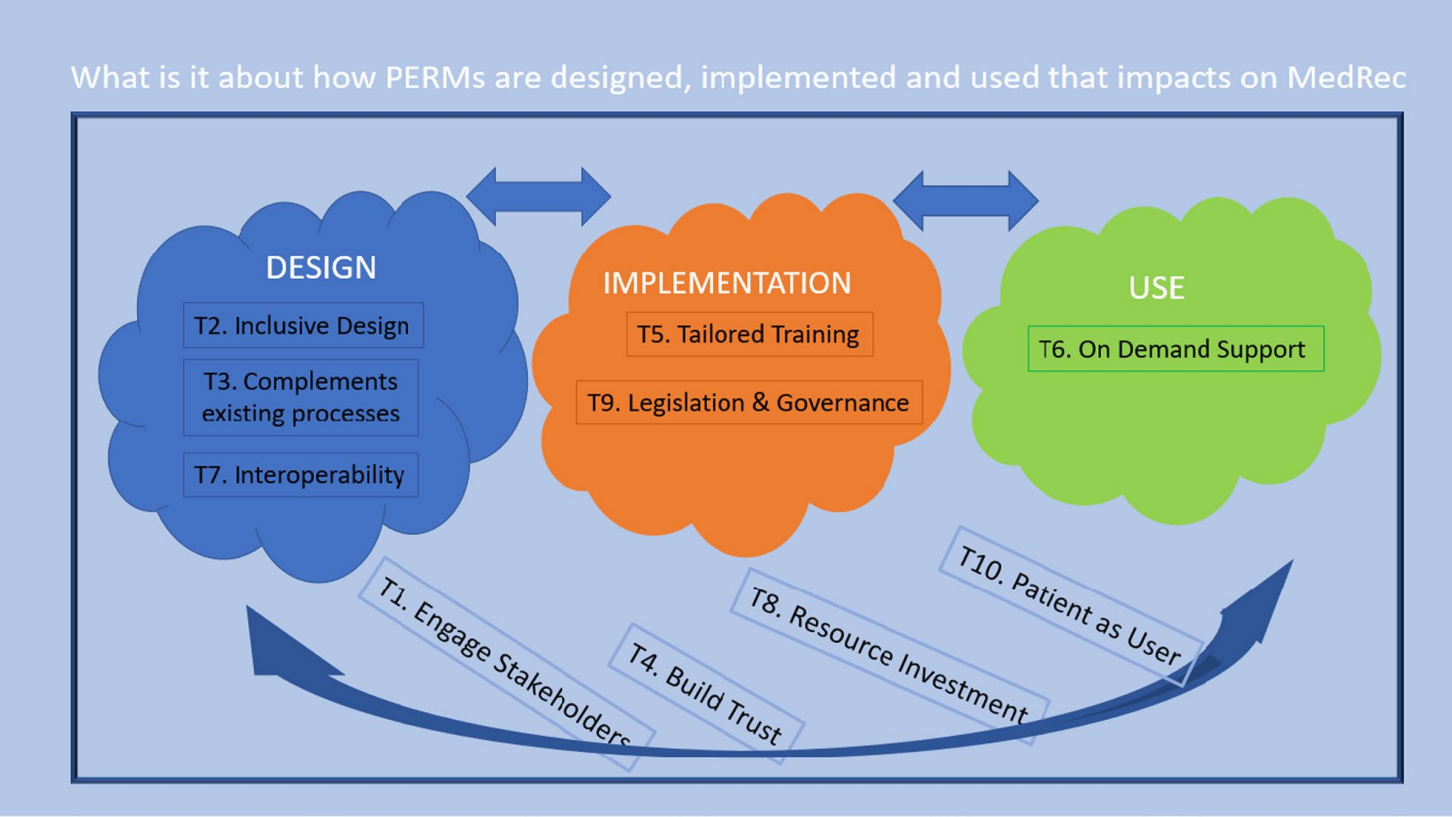
Theory framework in relation to phases of the PERM life-cycle

Some of the theories relate to the design (T2 Inclusive Design, T3 PERMs complement existing good processes, T7 Interoperability), some relate to the implementation (T5 Tailored Training, T9 Positive impact of Legislation or Governance), some relate to use (T6 Support and on-demand training) and many relate iteratively to all stages of the process (T1 Engage Stakeholders, T4 Build Trust, T8 Resource investment, T10 Patients as users of PERMs) (Fig. 3). Depending on the focus of the intervention or the stage in the development life-cycle, the range of theories incorporated will be different.

## Discussion

### Principal findings

We have developed ten theories to explain how, why, when, where and for whom PERMs are designed, implemented or used in practice at care transitions that impacts on MedRec based on data from 19 systematically sourced articles. These theories describe the contexts and mechanisms that impact on outcomes.

For the most part, the articles focused on two main outcomes: the patient’s safety and the user’s experience using a PERM. Only five studies referred to existing outcome frameworks or tools to assess the implementation or acceptance of the PERM [32, 35, 40, 45, 47]. Existing frameworks identified in the studies included the Technology Acceptance Model (TAM) [48], the Unified Theory of Acceptance and Use of Technology (UTAUT) [49], the Information Success Model [50] and The Clinical Adoption Framework [51]. Two articles reporting on the same intervention used the UTAUT and the DeLone & McLean models as the bases for their user satisfaction survey [40, 45], one used the themes from TAM during face-to-face interviews [32] and one used The Clinical Adoption Framework during their implementation process [35]. The other article referred to the various models in their background to the topic only [47]. Each of the above frameworks addresses elements of technology implementation generally, whilst our RRR specifically developed theories from evidence synthesis and stakeholder involvement to explain PERM design, implementation or use in practice at care transitions that impacts on MedRec.

Rahimi et al. [52] in their systematic review of the use of TAM in health informatics commented that its inconsistent predictive performance was related to the poor match between “construct operationalization and the context in which the construct is measured” [52]. By using a realist approach and mapping the broad contexts and mechanisms that influenced the design, implementation or use of PERMs to improve MedRec at care transitions identified in this review to Pawson et al.’s four I’s grouping of contexts outlined earlier in this article, we can hypothesise why the introduction of PERMs might succeed in one context and not in another:

- **Individuals** (characteristics and capacities of the various stakeholders):
  Consideration should be given to the individual user’s levels of confidence, trust, engagement and acceptance of PERMs. Users may have differing needs in relation to the amount, flexibility and format of training throughout the development and implementation life-cycle and therefore need differing levels of on-demand support in relation to both the MedRec process itself and the function and purpose of PERMs to support that process. Identification and comprehension of the user’s existing cognitive and decision-making processes and level of application of these to PERMs are important. For example, displaying pre-admission and in-hospital medication lists side by side on the screen reduces the cognitive burden and facilitates the task to compare these medication lists. An awareness of the changing roles of individuals undertaking MedRec is also important. For example, the expanding roles of the pharmacist and pharmacist technician.
- **Interpersonal relationships** (the stakeholder relationships that carry the intervention):
  The level of inclusive engagement of all stakeholders, including the individual users, in the design and implementation of PERMs; the level of awareness and respect for each other’s roles in the processes; and the level of clarity around who is responsible for the various stages of the MedRec processes, may all influence the successful introduction of PERMs.
- **Institutional settings** (the rules, norms and customs local to the intervention):
  The quality of the MedRec processes in place, the quality and complementarity of PERMs to support it and the complexity of the contexts in which PERMs will be used will influence success.
- **Infrastructure** (the wider social, economic and cultural setting of the intervention):
  The level of interoperability of systems supporting PERMs; the level of existing and future legislation and governance supporting MedRec and use of PERMs; the quality of the sources of data for MedRec; and the extent of resourcing including budgetary and human resources, required to allow PERMs to achieve their full potential, will influence success.

Many of the theories, if the term PERM was removed, could be applicable to any electronic system, including in a healthcare setting, where multiple users are using the system for different purposes.

However, focusing specifically on PERMs for MedRec at care transitions, Theory 3 states that for PERMs to become embedded into normal work practices they should complement an existing MedRec process. Some examples from the studies of the elements that could be complemented were: organisation and display of information following the natural order of events in a patient encounter; supporting workflow and accreditation requirements; medication information ordered by clinical importance; access to the history of a person’s previous medication use; inclusion of a function to prompt the user to record medication changes and reason for starting or stopping medications; inclusion of a field to capture details identified about a person’s medication adherence, allergies, or posologies; date of initiation of prescription and first prescriber; access to the prescribers’ or other HCPs’ contact details and the ability to produce a discharge summary and/or patient friendly print out.

Elements of PERMs, identified in the studies, that were expected to improve the frequency and integrity of MedRec, cognitive burden and decision making were: use of generic drug names thereby reducing confusion and duplication, organisation of information from different sources with the ability to see several lists at the same time facilitating rapid comparisons, information displays tailored to the needs of different users, ease of identifying medication changes, alerts i.e. drug-drug interactions or drug-disease interactions, reasonable balance of alerts, checks or warnings, action buttons (i.e. for stopping, modifying or continuing drugs), edit, sort (i.e. by therapeutic intent), drag and drop, or on-demand and additional information options, 24 h access to information, smooth processes to get patient consent to access their information. Barriers included number of clicks needed, too much text, unclear icons and lack of understanding or training in relation to how PERMs worked.

In relation to Theory 7, the quality of the data sources and level of interoperability between them, allowing safe and accurate sharing of information, was determined to be crucial to the successful introduction of PERMs.

Theory 8 identified that in order for the full potential of MedRec using PERMs to be realised, proper investment in the technology and sources of information must be provided, ensuring interoperability and access to accurate information for MedRec. For this to occur, increased patient safety and decreased hospital readmission rates and preventable events must be sufficiently valued.

### Comparisons with other literature

A number of systematic reviews have examined the use of electronic MedRec at care transitions [8, 13, 53–55]. Both Wang et al. and Mekonnen et al. reported equivocal findings regarding the impact of electronic MedRec tools on the prevalence of medication discrepancies or the proportion of patients experiencing (unintentional) discrepancies [13, 54]. Mekonnen reported that effective MedRec likely requires a multi-faceted approach involving people, process and technology, a finding which endorses the importance of our rapid realist review to identify CMOCs and generation of theories to test in the field. This multi-faceted approach should be addressed in future research. In their systematic review, Marien et al. compiled a list of recommendations for the successful development and implementation of electronic MedRec tools [8]. Although theirs was not a realist review, the recommendations reported mirror many of the contexts identified within our review, for example, development and implementation contexts, design features and functionalities. Our rapid realist review builds on this work by configuring contexts with mechanisms to influence a broad range of outcomes. Wang, Marien and Mekonnen all refer to the absence of evidence about usability, user satisfaction and user adherence [8, 13, 54]. Several of the theories generated in our review address these issues and future work to validate our theoretical framework in the field should therefore contribute to this evidence gap.

The scoping review by Monkman et al. examined both the contextual and human factors perspectives of using PERMs for MedRec and had several findings similar to those found in Bassi’s [53], Marien’s [8] and our review; successful implementation of electronic MedRec systems requires well designed systems, attention to implementation features and standardisation of the MedRec process [55]. Specifically identified were interoperability, design and layout of the system, clear MedRec processes and workflow, identification of who is responsible for MedRec and involvement of the user in the design especially for complex systems [8, 53, 55].

Comparing this review to the general health information technology (HIT) literature, Ammenwerth et al.’s framework focused on what they described as the “fit” of three key elements; individuals, task and technology (FITT framework) [56]. They identified what in realist terminology are the contexts and mechanisms of these three interacting elements with the aim of better understanding the reasons for information technology (IT) introduction failures. They identified that the user must be motivated, flexible and open to new ways of working; knowledgeable about the task and use of IT and trained to use the technology. The organisation must have a team culture and support the introduction of the HIT. The task must be organised; the complexity of the task to be completed must be considered. The technology must be functional, interoperable and usable. These are all consistent with the findings of this review and support theories 1. Engage Stakeholders, 2. Inclusive Design, 5. Tailored Training, 7. Interoperability and 10. Patients as users of PERMs.

Yen et al. considered how HIT implementation is evaluated. They suggest including evaluation of technology acceptance, communication and collaboration, work productivity, training and competency, leadership, existing policy, the organisational culture, the level of social support provided and the idiosyncrasies across contexts [57]. Marien et al. identified many reports lacked adequate information and recommended that study reports should carefully describe each component and that validated measures of usability should be reported [8]. The findings of our review, which included nine studies published since the Marien et al. review, are that such measures have still not been adopted. The importance of evaluating sociotechnical factors and usability outlined in these reviews are consistent with the finding of our review as outlined in theories 1. Engage Stakeholders, 2. Inclusive Design, 4. Build Trust, and 9. Positive impact of Legislation or Governance.

Shachak et al. described what the provided support for PERMs should look like, describing it under three headings; functional support: e.g. assistance in learning how to use the various features of the system; data support: e.g. activities intended to ensure the completeness, accuracy and consistency of data input and finally; training and education support, which they combined and stressed was an essential part of end-user support [58]. Such recommendations were re-iterated by Marien et al., specific to implementation of electronic MedRec tools [8]. This is consistent with the findings of this review specifically theories 5. Tailored Training and 6. Support and on-demand training.

The HIT literature features many of the contexts identified in this review as impacting on the design, implementation and use of PERMs and supports the validity of evaluating outcomes under the four I groupings referred to earlier as outlined by Pawson et al.; the individuals, interpersonal relationships, institutional settings and infrastructure [22, 56–58].

Acknowledging the associated human and financial resources required to deal with the increased effort, volume and quality of data gathered and generated when using PERMs for MedRec was identified in this review as being important. This finding was unique in the literature. The increased data potentially available provides opportunities for risk identification, management, and analysis; if the value of these opportunities is recognised by leaders/ management from the outset then they will understand the need for additional resources. (Theory 8. Resource investment). However, consistent with Sevick et al.*’s* [59] finding regarding electronic discharge communications, we identified the lack of evidence and the importance of evaluating the cost effectiveness of using PERMs for MedRec. Until such evidence is available, convincing individual organisations or countries of the additional benefits will be difficult. Careful consideration of the outcomes to be assessed in any cost analysis is also needed [59].

### Strengths and limitations

A RRR, by its nature does not include all relevant literature on the topic of interest, however, we strived to include the richest data in this review, and the reviewers feel that saturation for each of the theories was achieved. If time allowed, a specific search through the remaining 32 articles included in the full text reading for other potential contexts and mechanisms identified by the panels during the review of the final theories, but lacking evidence in the 19 included articles, could have provided enough evidence to support the development of additional theories. Examples of such contexts include organisational culture or level of reliance on PERMs. We recommend this as a future study.

The quality assessment of realist data, using relevance, rigour and richness, could be considered a subjective assessment of the quality of the data within the articles included. However, the research team have extensive knowledge and experience in the field under review allowing expert assessment of these elements. We followed the RAMESES guidelines for the quality assessment process by providing a transparent procedure for the rating systems used to appraise the evidence used within the review; the real quality has been determined within the act of synthesis [60]. The iterative nature of realist reviews resulted in the richness rating, the method used to focus the review, for some articles being revised downwards at the data extraction stage.

This RRR has allowed additional valuable data to be extracted from existing primary research, with minimal resources, that may impact positively on the future design, implementation and use of PERMs at care transitions for MedRec and of research in this area. The main outcomes formally considered in the included articles focused on either the impact on patient safety or the user’s experiences during implementation or use of a PERM. The use of realist methods has generated data to support identification of additional outcomes from the articles which were not formally identified by the authors in their aims and objectives. The additional outcomes included changes to interprofessional relationships, changes to awareness of others’ roles, changes to the users’ cognitive burden, changes to the consent process when sharing patient information, changes to the level of interaction with patients, changes to the patients level of awareness regarding the need for their consent, their opinions in relation to the benefits of interoperability/sharing their information and their level of engagement with PERMs. Consideration of this evidence has added richness to the knowledge identified in the systematic reviews referred to earlier, validating our chosen methodology. Future research should include appraisal of these socio-technical outcomes.

### Practical implications

The product of realist reviews are theories, developed from evidence extracted from the literature and the input of experts in the field. These theories should subsequently be incorporated into an intervention or mapped to existing interventions and evaluated to further test their validity and refined or rejected based on that evaluation.

## Conclusions

The use of realist methodology to investigate what is it about how, why, when, where and for whom PERMs are designed, implemented or used in practice at care transitions that impacts on medical reconciliation, has proved efficient and effective. We developed ten theories that identified the contexts and mechanism that may impact on the successful introduction of PERMs at care transitions for MedRec. Engaging all stakeholders; allowing a free flow of ideas and feedback; building trust in relation to the accuracy, safety and security of the data; providing sufficient resources for the full potential of PERMs to be realised; considering interoperability; and the value of patients having a role in using PERMs for MedRec, were considered important elements at all stages of the process.

Further research to assess the application of these theories in practice is now required. Future research in this area must also include evaluation of all aspects and at all stages of the process of introducing PERMs, including sociotechnical factors and cost analysis. Further realist reviews could be undertaken to examine other elements that may impact on the use of MedRec at care transitions that this RRR did not cover, such as organisational culture and level of reliance on PERMs.

## Supplementary Information

**Additional file 1**. Protocol.

**Additional file 2**. Search terms.

**Additional file 3**. Inclusion and exclusion criteria.

**Additional file 4**. Themes identified by the Reference Panel.

**Additional file 5**. NVIVO codebook.

**Additional file 6**. Template for summary of study document.

**Additional file 7**. The supporting contexts, mechanisms and outcomes configurations.

**Additional file 8**. Introduction for expert and reference panels.

**Additional file 9**. Feedback survey.

**Additional file 10**. List of included studies.

**Additional file 11**. Feedback responses.

**Additional file 12**. Full data synthesis.

## Data Availability

All data generated or analysed during this study are included in this published article and its additional files.

## Abbreviations

CMOCs: Context Mechanism Outcome Configurations
HIT: Health Information Technology
IT: Information Technology
MedRec: Medication Reconciliation
MRP: Medicine-related problems
PERMs: Personal Electronic Records of Medicines
RAMESES: Realist And Meta-narrative Evidence Syntheses: Evolving Standards
