# Supplementary material for: Personal Electronic Records of Medications (PERMs) for medication reconciliation at care transitions: a rapid realist review
Source: BMC Med Inform Decis Mak. 2021 Nov 3;21:307. doi: 10.1186/s12911-021-01659-8 (PMC8565006; doi:10.1186/s12911-021-01659-8)
Supplement: Supplementary file 1 — Additional file 1. Protocol. [file 12911_2021_1659_MOESM1_ESM.pdf]

Systematic review

*Fields that have an **asterisk (*)** next to them means that they **must be answered. Word limits provide guidance** but do not actually limit the number of words that can be entered in each section. You are encouraged to follow maximum length. Registrant means the person filling out the form.*

1. * Review title.

Give the title of the review in English

What is it about how, why, when and for whom Personal Electronic Records of Medications (PERM) are designed, implemented or used in practice at care transitions that impacts on medical reconciliation; A Rapid Realist Review?

2. Original language title.

For reviews in languages other than English, give the title in the original language. This will be displayed with the English language title.

3. * Anticipated or actual start date.

Give the date the systematic review started or is expected to start.

04/08/2020

4. * Anticipated completion date.

Give the date by which the review is expected to be completed.

04/02/2021

5. * Stage of review at time of this submission.

Tick the boxes to show which review tasks have been started and which have been completed.
Update this field each time any amendments are made to a published record.

Reviews that have started data extraction (at the time of initial submission) are not eligible for inclusion in PROSPERO.
If there is later evidence that incorrect status and/or completion date has been supplied, the published PROSPERO record will be marked as retracted.

This field uses answers to initial screening questions. It cannot be edited until after registration.

The review has not yet started: No

Review stage	Started	Completed
Preliminary searches	Yes	Yes
Piloting of the study selection process	Yes	Yes
Formal screening of search results against eligibility criteria	No	No
Data extraction	No	No
Risk of bias (quality) assessment	No	No
Data analysis	No	No

Provide any other relevant information about the stage of the review here.

Protocol being used is that of a rapid realist review.
Protocol being used is that of a rapid realist review.

6. * Named contact.

The named contact is the guarantor for the accuracy of the information in the register record. This may be any member of the review team.

Tamasine Grimes

Email salutation (e.g. "Dr Smith" or "Joanne") for correspondence:
Dr Grimes

7. * Named contact email.

Give the electronic email address of the named contact.

TAGRIMES@tcd.ie

8. Named contact address

PLEASE NOTE this information will be published in the PROSPERO record so please do not enter private information, i.e. personal home address

Give the full institutional/organisational postal address for the named contact.

School of Pharmacy, Panoz Institute, Trinity College Dublin, Dublin 2

9. Named contact phone number.

Give the telephone number for the named contact, including international dialling code.

+353 1 896 2805

10. * Organisational affiliation of the review.

Full title of the organisational affiliations for this review and website address if available. This field may be completed as 'None' if the review is not affiliated to any organisation.

Trinity College Dublin

Organisation web address:
www.tcd.ie

11. * Review team members and their organisational affiliations.

Give the personal details and the organisational affiliations of each member of the review team. Affiliation refers to groups or organisations to which review team members belong.
NOTE: email and country now MUST be entered for each person, unless you are amending a published record.

Dr Tamasine Grimes. School of Pharmacy, Trinity College Dublin
Dr Tim Delaney. Tallaght University Hospital, Dublin
Assistant/Associate Professor Sam Cromie. Centre for Innovative Human Studies, TCD
Dr Joan Cahill. Centre for Innovative Human Studies, TCD
Assistant/Associate Professor Sean Kennelly. Tallaght University Hospital and TCD
Assistant/Associate Professor Joshua Pevnick. Cedars-Sinai, Los Angeles, California, USA
Dr Catherine Waldron. School of Pharmacy, Trinity College Dublin

12. * Funding sources/sponsors.

Details of the individuals, organizations, groups, companies or other legal entities who have funded or sponsored the review.

The Meath Foundation Research Grant 2019

Grant number(s)
State the funder, grant or award number and the date of award

Not applicable

13. * Conflicts of interest.

List actual or perceived conflicts of interest (financial or academic).

None

14. Collaborators.

Give the name and affiliation of any individuals or organisations who are working on the review but who are not listed as review team members. **NOTE: email and country must be completed for each person, unless you are amending a published record.**

Professor Neil O'Hare. Chief Information Officer for Ireland East Hospital Group, Prof. of Health Informatics University College Dublin (UCD)

Mr Brian Markey. Senior Project Manager - National ePrescribing Project, Health Service Executive (HSE)

Dr Rosa McNamara. Consultant physician, St James Hospital

Ms Roisin Adams. Head of HTA Strategy and External engagement, National Centre for Pharmacoeconomic

Mr Kevin O'Carroll. Technical Standards Manager, Health Information and Quality Authority (HIQA)

Ms Louise McQuaid. Technical Standards, HIQA

Ms Ciara Kirke. Clinical Lead, Medication Safety, National Quality Improvement Team, HSE

Ms Muriel Pate. Medication Safety Specialist Pharmacist, National Quality Improvement Team, HSE

Mr Dan Burns. Community Pharmacist

Mr Alan Reilly. Head of Information and Technology, Irish Pharmacy Union

Ms Jane Kenny. Clinical Pharmacy Services Manager, Tallaght University Hospital, Tallaght

Dr Tora Hammar. Linnaeus University, Dept. of Health and Life Sciences

Dr Patrick Redmond.

Dr Ann Slee. National Health Service

Mr Andrew Jones. Subject Librarian TCD.

15. * Review question.

State the review question(s) clearly and precisely. It may be appropriate to break very broad questions down into a series of related more specific questions. Questions may be framed or refined using PI(E)COS or similar where relevant.

In what circumstances is the use of Personal Electronic Records of Medications (PERM) in care transition most likely to be effective?

16. * Searches.

State the sources that will be searched (e.g. Medline). Give the search dates, and any restrictions (e.g. language or publication date). Do NOT enter the full search strategy (it may be provided as a link or attachment below.)

Searches will be limited to the English language

Databases to be searched: PubMed, Embase, CINAHL Complete, OpenGrey

The reference lists of relevant articles (chaining) and snowballing will also be carried out.

Purposeful searching, particularly for qualitative reports, interviews or surveys and reports of negative findings will continue throughout the review. All article types will be eligible for inclusion, for example, policy documents, newspaper articles and opinion pieces, no study designs will be excluded. Relevant review articles will be searched for relevant articles not already included, if numbers are low.

Full text review of potentially relevant articles will be completed. A subsample screening of the full texts will be undertaken by two screeners to ensure consistency, and disagreements will be resolved by a 3rd reviewer.

The search will have two phases; the first phase will focus specifically on medication reconciliation using PERM at care transitions, the second phase, depending on the quality and quantity of the findings of the first phase, will either extend the search to include medication reconciliation using PERM at times other than at care transition or narrow the focus to a type of PERM or particular patient cohort.

17. URL to search strategy.

Upload a file with your search strategy, or an example of a search strategy for a specific database, (including the keywords) in pdf or word format. In doing so you are consenting to the file being made publicly accessible.

Or provide a URL or link to the strategy. Do NOT provide links to your search **results**.

Do not make this file publicly available until the review is complete

18. * Condition or domain being studied.

Give a short description of the disease, condition or healthcare domain being studied in your systematic review.

Medication reconciliation using personal electronic records of medications at care transitions

19. * Participants/population.

Specify the participants or populations being studied in the review. The preferred format includes details of both inclusion and exclusion criteria.

All persons involved in the reconciliation of medications at care transition

20. * Intervention(s), exposure(s).

Give full and clear descriptions or definitions of the interventions or the exposures to be reviewed. The preferred format includes details of both inclusion and exclusion criteria.

Any intervention involving medication reconciliation using PERM at care transition.
Definition of Terms:
Medication Reconciliation: Any opportunity taken to compare one list of medications with another
Care Transition: Any movement between care settings or change in responsibility of care of a patient
PERM: Any electronic system used to record and store information regarding the medications (past or current) prescribed, dispensed or used by patients that may contribute to a record of their medication history.
(Any articles reporting medication reconciliation using PERM at times other than at care transition will be tagged in case numbers are low)

21. * Comparator(s)/control.

Where relevant, give details of the alternatives against which the intervention/exposure will be compared (e.g. another intervention or a non-exposed control group). The preferred format includes details of both inclusion and exclusion criteria.

Include studies with and without comparator in our review.

22. * Types of study to be included.

Give details of the study designs (e.g. RCT) that are eligible for inclusion in the review. The preferred format includes both inclusion and exclusion criteria. If there are no restrictions on the types of study, this should be stated.

There will be no restrictions placed on the type of study design used in the inclusion criteria as per Realist Methodology

23. Context.

Give summary details of the setting or other relevant characteristics, which help define the inclusion or exclusion criteria.

Transition within or between care settings, no restriction on care setting. Any article that suggests that there might be information of value to the review in relation to the interplay between stakeholders during design, implementation and use of PERM or the stakeholders' responses/reactions to the resources and opportunities provided by PERM. Qualitative articles where the opinions of the stakeholders in a particular setting has been sought or applied in practice and articles reporting negative findings in relation to these elements in practice are important

24. * Main outcome(s).

Give the pre-specified main (most important) outcomes of the review, including details of how the outcome is defined and measured and when these measurement are made, if these are part of the review inclusion criteria.

The primary outcome of this rapid realist review is to develop informed theories about what, how, why, when and for whom medication reconciliation using PERM at care transitions are effective or not. The exact outcomes will be dependent on the evidence that exists in the literature. Some likely examples of the “what, how, why, when and for whom” questions that we hope will be answered are:

What: What content was available on the PERM? What training was provided to use the PERM? What integration with work systems or workflows occurred?

How: How was the PERM designed? How safe was the information? How was the PERM implemented in a particular setting? How frequently is the PERM used? How were the impacts (positive or negative) monitored or evaluated?

Why: Why was a PERM successfully implemented in one setting and not another?

When: Does the timing of implementation of the PERM impact on its use in practice.

For Whom: Who needs information from PERM in practice? Who uses PERM?

* Measures of effect

N/A

25. * Additional outcome(s).

List the pre-specified additional outcomes of the review, with a similar level of detail to that required for main outcomes. Where there are no additional outcomes please state ‘None’ or ‘Not applicable’ as appropriate to the review

None

* Measures of effect

N/A

26. * Data extraction (selection and coding).

Describe how studies will be selected for inclusion. State what data will be extracted or obtained. State how this will be done and recorded.

The screening of the titles and abstracts will be completed by at least two reviewers independently using the inclusion and exclusion criteria outlined. A pilot of the screening process will be undertaken to clarify the inclusion and exclusion criteria.

The full text articles will be sourced, and the data extracted by one reviewer (CW). The NVIVO software programme will be used to manage the full texts of the included papers. A random subsection (10%) of the full text articles will be reviewed and data extracted by additional reviewers (TG, SC and/or JC) early in the process and any disagreement will be resolved by discussion before continuing any further data extraction. Relevant sections of the texts will be coded under the broad headings (Nodes) of Contexts, Mechanisms and Outcomes with subheadings based on the themes developed by the Reference Panel and on any additional new concepts identified in the literature. This is an iterative process and so steps may need to be repeated as new concepts come to light.

Depending on the number of relevant papers and the time limit for the process, some focusing may be required. A detailed record will be kept of all decisions and the justification for them in relation to any focusing of the review and will be guided by the opinions of the expert panel and the feasibility of applying the theories in practice.

27. * Risk of bias (quality) assessment.

State which characteristics of the studies will be assessed and/or any formal risk of bias/quality assessment tools that will be used.

Relevance – Does the article provide information of value to the review in relation to the interplay between stakeholders during design, implementation and use of PERM or the stakeholders’ responses/reactions to the resources and opportunities provided by PERM.

Rigour – Are the sources or methods used to generate the relevant data credible and trustworthy.

Assessment of relevance and rigour will be undertaken during the extraction process and will be based on the individual sections of extracted data rather than the overall article, as per RAMESES guidelines. This may include discussion/debate within the review team of the findings or consultation with experts about technical aspects.

28. * Strategy for data synthesis.

Describe the methods you plan to use to synthesise data. This **must not be generic text** but should be **specific to your review** and describe how the proposed approach will be applied to your data.

If meta-analysis is planned, describe the models to be used, methods to explore statistical heterogeneity, and software package to be used.

The strategy for the data synthesis will be guided by the RAMESES Standards of reporting for Realist Reviews. A realist "philosophical lens" as described in the Standards is required and this makes it very different to other methods of data synthesis. The purpose of the analysis is not simply to be descriptive but to be explanatory. The question is not if an intervention works or not, the review seeks to explain how and why interventions generate different outcomes in different contexts. The analytical task is to find and align the evidence to demonstrate that particular mechanisms generate particular outcomes and to identify which elements of the context matter; the CMO configurations (CMOCs).

The following analytical processes based on Pawson's Evidence Based Policy, will be used at all stages of the review to make sense of the CMOCs being developed:

a) Juxtaposing – where evidence about mechanisms in one source enables insights into outcome patterns of another source.

b) Reconciling – finding explanations for different outcomes by uncovering contextual differences.

c) Adjudication – explaining opposing study outcomes on the basis of methodological strengths and weaknesses.

d) Consolidation – where outcomes differ in particular contexts and explanations can be constructed of how and why these differences occurred.

e) Situating – describing which mechanisms were activated in which context.

The end result should be series of statements or theories based on the literature examined which describe what is it about how, why, when and for whom Personal Electronic Records of Medications (PERM) are designed, implemented or used in practice at care transitions that impacts on medical reconciliation.

29. * Analysis of subgroups or subsets.

State any planned investigation of ‘subgroups’. Be clear and specific about which type of study or participant will be included in each group or covariate investigated. State the planned analytic approach.

None planned

30. * Type and method of review.

Select the type of review, review method and health area from the lists below.

Type of review

Cost effectiveness	No
Diagnostic	No
Epidemiologic	No
Individual patient data (IPD) meta-analysis	No
Intervention	No
Meta-analysis	No
Methodology	No
Narrative synthesis	No
Network meta-analysis	No
Pre-clinical	No
Prevention	No
Prognostic	No
Prospective meta-analysis (PMA)	No
Review of reviews	No
Service delivery	No

Synthesis of qualitative studies	No
Systematic review	Yes
Other	Yes

A Rapid Realist Review

Health area of the review

Alcohol/substance misuse/abuse	No
Blood and immune system	No
Cancer	No
Cardiovascular	No
Care of the elderly	No
Child health	No
Complementary therapies	No
COVID-19	No
Crime and justice	No
Dental	No
Digestive system	No
Ear, nose and throat	No
Education	No
Endocrine and metabolic disorders	No
Eye disorders	No
General interest	No
Genetics	No
Health inequalities/health equity	No
Infections and infestations	No
International development	No
Mental health and behavioural conditions	No
Musculoskeletal	No
Neurological	No
Nursing	No
Obstetrics and gynaecology	No

Oral health	No
Palliative care	No
Perioperative care	No
Physiotherapy	No
Pregnancy and childbirth	No
Public health (including social determinants of health)	Yes
Rehabilitation	No
Respiratory disorders	No
Service delivery	No
Skin disorders	No
Social care	No
Surgery	No
Tropical Medicine	No
Urological	No
Wounds, injuries and accidents	No
Violence and abuse	No

31. Language.

Select each language individually to add it to the list below, use the bin icon to remove any added in error.

English

There is not an English language summary

32. * Country.

Select the country in which the review is being carried out. For multi-national collaborations select all the countries involved.

Ireland

33. Other registration details.

Name any other organisation where the systematic review title or protocol is registered (e.g. Campbell, or The Joanna Briggs Institute) together with any unique identification number assigned by them.
If extracted data will be stored and made available through a repository such as the Systematic Review Data Repository (SRDR), details and a link should be included here. If none, leave blank.

34. Reference and/or URL for published protocol.

If the protocol for this review is published provide details (authors, title and journal details, preferably in Vancouver format)

No I do not make this file publicly available until the review is complete

35. Dissemination plans.

Do you intend to publish the review on completion?

Yes

We planned to publish the findings of this Review.

36. Keywords.

Give words or phrases that best describe the review. Separate keywords with a semicolon or new line. Keywords help PROSPERO users find your review (keywords do not appear in the public record but are included in searches). Be as specific and precise as possible. Avoid acronyms and abbreviations unless these are in wide use.

Medication Reconciliation, Care transition, Electronic Health Records, Health Information Exchange

37. Details of any existing review of the same topic by the same authors.

If you are registering an update of an existing review give details of the earlier versions and include a full bibliographic reference, if available.

None

38. * Current review status.

Update review status when the review is completed and when it is published.
New registrations must be ongoing.

Review_Ongoing

39. Any additional information.

Provide any other information relevant to the registration of this review.

40. Details of final report/publication(s) or preprints if available.

Leave empty until publication details are available OR you have a link to a preprint.
List authors, title and journal details preferably in Vancouver format.
