# Supplementary material for: Personal Electronic Records of Medications (PERMs) for medication reconciliation at care transitions: a rapid realist review
Source: BMC Med Inform Decis Mak. 2021 Nov 3;21:307. doi: 10.1186/s12911-021-01659-8 (PMC8565006; doi:10.1186/s12911-021-01659-8)
Supplement: Supplementary file 2 — Additional file 2. Search terms. [file 12911_2021_1659_MOESM2_ESM.pdf]

Additional File 2: Search Strategy

PubMed 01/09/2020

All Fields, Filters: English, Human, Abstract

Search: ("Continuity of Patient Care" OR "Care Continuity" OR "Patient Care Continuity" OR "Continuum of Care" OR "Care Continuum" OR "Continuity of Care" OR "Interdisciplinary communication" OR "Transitional care" OR "Patient Discharge" OR "care transition" OR "care transitions" OR "Community hospital-link") AND ("Medication therapy management" OR "Medication Reconciliation" OR "Child medication reconciliation" OR "Elderly medication reconciliation" OR "Medication Accuracy" OR "eMedRec" OR "MedRec" OR "Med Rec" OR "Electronic medication reconciliation" OR "Medication history taking" OR "Best possible medication history" OR "Best possible medication list" OR "Gold standard patient medication list" OR "Gold standard pre admission patient medication list" OR "Medication list" OR "Medication histories" OR "Medication history" OR "Medication Error" OR "Drug Use Error" OR "Drug discrepancy" OR "Unintentional discrepancy" OR "Medication discrepancy" OR "Prescription discrepancy" OR "Unintentional medication discrepancy" OR "Adverse drug event" OR "Adverse medication event" OR "Drug omission" OR "Drug duplication" OR "Drug dosing errors" OR "Medication omission" OR "Medication duplication" OR "Medication dosing errors" OR "Medication discrepancies" OR "Medication Safety" OR "Medication Accuracy" OR "drug omission") AND ("Electronic health record" OR "Personal Electronic Health Records" OR "Electronic Health Records" OR "Computerised Patient Record" OR "Health information exchange" OR "Medical informatics" OR "Electronic health records" OR "Drugs information system" OR "Computerised health record" OR "Computerised medication record" OR "Computerised drug history" OR "Electronic drug history" OR "Computerized Patient Medical Records" OR "Automated Medical Records System" OR "Computerized Medical Record System" OR "Automated Medical Record System" OR "Electronic Medical Records" OR "Electronic Medical Record" OR "Electronic Health Record" OR "Computerized Medical Record" OR "Medication list" OR "Dispensing list" OR "Prescribing list" OR "Prescription drug database" OR "Prescription database" OR "Pharmacy record" OR "Pharmacy records" OR "Pharmacy claim" OR "Pharmacy claims" OR "Pharmacy claims data" OR "Pharmacy claims database" OR "Prescription data" OR "Prescription database" OR "Dispensing data" OR "Dispensing database" OR "Dispensing record" OR "Dispensing repository" OR "Prescription repository" OR "Pharmacy claims repository" OR "Medication list repository" OR "Dispensing list repository" OR "Pharmacy record repository" OR "Drug history repository" OR "Drug record repository" OR "Medication history repository" OR "Medication record repository" OR "Shared care record" OR "Health Information Interoperability" OR "interoperability" OR "User-Computer Interface" OR "Human computer interaction" OR "human machine interaction" OR "user experience" OR "information behaviour" OR "information management" OR "information use" OR "information need" OR "human factors") Filters: Abstract

EMBASE 01/09/2020

Quick Search, All fields, Filters English, Human, Abstracts

('continuity of patient care' OR 'patient care continuity' OR 'continuum of care' OR 'care continuum' OR 'continuity of care' OR 'care continuity' OR 'interdisciplinary communication' OR 'transitional care' OR 'patient discharge' OR 'care transition' OR 'care transitions' OR 'community hospital-link')

AND

('medication therapy management' OR 'medication reconciliation' OR 'child medication reconciliation' OR 'elderly medication reconciliation' OR 'emedrec' OR 'medrec' OR 'med rec' OR 'electronic medication reconciliation' OR 'medication history taking' OR 'best possible medication history' OR 'best possible medication list' OR 'gold standard patient medication list' OR 'gold standard pre admission patient medication list' OR 'medication list' OR 'medication histories' OR 'medication history' OR 'medication error' OR 'drug use error' OR 'drug discrepancy' OR 'unintentional discrepancy' OR 'medication discrepancy' OR 'prescription discrepancy' OR 'unintentional medication discrepancy' OR 'adverse drug event' OR 'adverse medication event' OR 'drug duplication' OR 'drug dosing errors' OR 'medication omission' OR 'medication duplication' OR 'medication dosing errors' OR 'medication discrepancies' OR 'medication safety' OR 'medication accuracy' OR 'drug omission')

AND

('personal electronic health records' OR 'computerised patient record' OR 'health information exchange' OR 'medical informatics' OR 'electronic health records' OR 'drugs information system' OR 'computerised health record' OR 'computerised medication record' OR 'computerised drug history' OR 'electronic drug history' OR 'computerized patient medical records' OR 'automated medical records system' OR 'computerized medical record system' OR 'automated medical record system' OR 'electronic medical records' OR 'electronic medical record' OR 'electronic health record' OR 'computerized medical record' OR 'medication list' OR 'dispensing list' OR 'prescribing list' OR 'prescription drug database' OR 'pharmacy record' OR 'pharmacy records' OR 'pharmacy claim' OR 'pharmacy claims' OR 'pharmacy claims data' OR 'pharmacy claims database' OR 'prescription data' OR 'prescription database' OR 'dispensing data' OR 'dispensing database' OR 'dispensing record' OR 'dispensing repository' OR 'prescription repository' OR 'pharmacy claims repository' OR 'medication list repository' OR 'dispensing list repository' OR 'pharmacy record repository' OR 'drug history repository' OR 'drug record repository' OR 'medication history repository' OR 'medication record repository' OR 'shared care record' OR 'health information interoperability' OR 'interoperability' OR 'user-computer interface' OR 'human computer interaction' OR 'human machine interaction' OR 'user experience' OR 'information behaviour' OR 'information management' OR 'information use' OR 'information need' OR 'human factors') AND [humans]/lim AND [english]/lim AND [abstracts]/lim

CINAHL 01/09/2020

Limiters - Abstract Available; English Language; Human

Expanders - Apply equivalent subjects

Search modes - Boolean/Phrase

AB ((“Continuity of Patient Care” OR “Care Continuity” OR “Patient Care Continuity” OR “Continuum of Care” OR “Care Continuum” OR “Continuity of Care” OR “Interdisciplinary communication” OR “Transitional care” OR “Patient Discharge” OR “care transition” OR “care transitions” OR "Community hospital-link"))

AND

AB (("Medication therapy management" OR "Medication Reconciliation" OR “Child medication reconciliation” OR “Elderly medication reconciliation” OR “Medication Accuracy” OR “eMedRec” OR “MedRec” OR “Med Rec” OR “Electronic medication reconciliation” OR “Medication history taking”

OR “Best possible medication history” OR “Best possible medication list” OR “Gold standard patient medication list” OR “Gold standard pre admission patient medication list” OR “Medication list” OR “Medication histories” OR “Medication history” OR “Medication Error” OR “Drug Use Error” OR “Drug discrepancy” OR “Unintentional discrepancy” OR “Medication discrepancy” OR “Prescription discrepancy” OR “Unintentional medication discrepancy” OR “Adverse drug event” OR “Adverse medication event” OR “Drug omission” OR “Drug duplication” OR “Drug dosing errors” OR “Medication omission” OR “Medication duplication” OR “Medication dosing errors” OR “Medication discrepancies” OR “Medication Safety” OR “Medication Accuracy” OR “drug omission”))

AND

AB (("Electronic health record" OR "Personal Electronic Health Records" OR "Electronic Health Records" OR "Computerised Patient Record" OR “Health information exchange” OR “Medical informatics” OR “Electronic health records” OR “Drugs information system” OR “Computerised health record” OR “Computerised medication record” OR “Computerised drug history” OR “Electronic drug history” OR “Computerized Patient Medical Records” OR “Automated Medical Records System” OR “Computerized Medical Record System” OR “Automated Medical Record System” OR “Electronic Medical Records” OR “Electronic Medical Record” OR “Electronic Health Record” OR “Computerized Medical Record” OR “Medication list” OR “Dispensing list” OR “Prescribing list” OR “Prescription drug database” OR “Prescription database” OR “Pharmacy record” OR “Pharmacy records” OR “Pharmacy claim” OR “Pharmacy claims” OR “Pharmacy claims data” OR “Pharmacy claims database” OR “Prescription data” OR “Prescription database” OR “Dispensing data” OR “Dispensing database” OR “Dispensing record” OR “Dispensing repository” OR “Prescription repository” OR “Pharmacy claims repository” OR “Medication list repository” OR “Dispensing list repository” OR “Pharmacy record repository” OR “Drug history repository” OR “Drug record repository” OR “Medication history repository” OR “Medication record repository” OR “Shared care record” OR "Health Information Interoperability" OR "interoperability" OR "User-Computer Interface" OR "Human computer interaction" OR "human machine interaction" OR “user experience” OR “information behaviour” OR “information management” OR “information use” OR “information need” OR “human factors”))

OpenGrey 01/09/2020

Limiters; English language

abstract AND human AND link;

“electronic patient record” OR “Summary Care Record” OR “Centralised electronic databases” OR “Aggregate patient medication records” OR “Aggregate patient medication records” OR “Centralised patient medication records” OR “Electronic Health Record” OR “National Shared Record” OR “Shared Summary Care Record” OR “Shared Care Record” OR “Individual Health Record” OR “Emergency Care Summary Record” OR “shared electronic patient record” OR “discharge summary” lang:"en"

As many of the OpenGrey articles were obviously not relevant to the review they were screened by title, and availability of an abstract for inclusion for screening. 55 reduced to 18 which were added to Covidence for review.
