# Supplementary material for: Personal Electronic Records of Medications (PERMs) for medication reconciliation at care transitions: a rapid realist review
Source: BMC Med Inform Decis Mak. 2021 Nov 3;21:307. doi: 10.1186/s12911-021-01659-8 (PMC8565006; doi:10.1186/s12911-021-01659-8)
Supplement: Supplementary file 3 — Additional file 3. Inclusion and exclusion criteria. [file 12911_2021_1659_MOESM3_ESM.pdf]

Additional File 3: Inclusion and Exclusion Criteria

- for Title and Abstract Screening and Full Text Screening

Title and Abstract Screening:

Inclusion criteria

The article must **broadly** relate to medication reconciliation using PERM at care transition.

Definition of Terms:

Medication Reconciliation: Any opportunity taken to compare one list of medications with another

Care Transition: Any movement between care settings or change in responsibility of care of a patient

PERM: Any electronic system used to record and store information regarding the medications (past or current) prescribed, dispensed or used by patients that may contribute to a record of their medication history.

(Any articles reporting medication reconciliation using PERM at times other than at care transition should be tagged in case numbers are low)

Type of Report: All article types are eligible including, for example, review articles, policy documents, newspaper articles and opinion pieces, the report does not need to be an intervention, no study designs are excluded.

Relevance – Any article that suggests that there might be information of value to the review in relation to the interplay between stakeholders during design, implementation and use of PERM or the stakeholders' responses/reactions to the resources and opportunities provided by PERM.

The articles should focus on medication reconciliation at care transition AND use of PERM. Qualitative articles where the opinions of the stakeholders in a particular setting has been sought or applied in practice and articles reporting negative findings in relation to these elements in practice are important.

Rigour - the sources or methods used to generate the relevant data are credible and trustworthy.

Exclusion criteria for Title and Abstract Screening:

Exclude articles not in the English language

Exclude if no abstract is available

Clarifications arising from the Pilot of Title and Abstract

1. All three elements must be present in the title or abstract (Med Rec, Care Transition and PERM) for inclusion. (Any articles reporting two elements that are felt to contain information relevant to the review should be tagged in case numbers are low, I have set up three separate tags for these, No MedRec, but interesting, No Care Transition but interesting, No PERM but interesting, then excluded)
2. Explicit mention of use of an electronic record – if a system is referred to (i.e PharmaNet, MARQUIS toolkit, SafeMed Programme) but the screener is unclear if this is an electronic system. Decision: choose Maybe.

If there is a suggestion of technology being used (i.e. “Emerging technologies”). Decision: choose Maybe.

If there is absolutely no mention or suggestion of an electronic record or technology being used. Decision: Tag it (Possible PERM) and see what the final number of included articles is.

3. If the article only reports the use of the electronic record to audit the Med Rec, but it’s use at care transition was not mentioned (Thorsteinsdottir 2015). Decision: Include for now.

4. If the article only reports the use of the electronic record to source the patient. Decision: Include for now.

5. Clarification of what “electronic record” means. Decision: a means to electronically store and share patient information. So includes a mobile phone but does not include a fax.

6. Review articles – if the topic of the review relates to any of the key topics (Med Rec, Care Transition or Use of electronic records). Decision: Tag (Review) and exclude. If numbers are low, this article will be screened for the included studies. The review itself will not be included in the review.

7. Should protocols be included? Decision: Tag and exclude, these will be followed up if numbers are low.

8. Don’t screen an article if you are an author.

Full Text Screening

<u>Inclusion Criteria</u>

Any article that assesses the facilitators or barriers to the use of PERM for MedRec in care transition

Able to shed light on any aspect of Context, Mechanism or Outcome for any element of the research questions/candidate theories

Focus on elements of any studies that relate to how they worked or not, as well as the outcome

The article may not necessarily be about the whole research question, but relevant to a sub-section of it (i.e. how HCP engage with technology)

<u>Exclusion Criteria</u>

Exclude any article that relates **only** to medication errors resulting from causes other than those relating to care transition (i.e. drug interaction, compliance/adherence, non-electronic records).

Exclude any article that relates **only** to reports of the use of technological tools/algorithms to resolve medication reconciliation issues, i.e. does not include contexts or human-computer interface elements.

Exclude any article that seeks out relevant data from situations outside the intervention under study where it can be reasonably inferred that the same mechanisms(s) might be in operation.
