# Supplementary material for: Personal Electronic Records of Medications (PERMs) for medication reconciliation at care transitions: a rapid realist review
Source: BMC Med Inform Decis Mak. 2021 Nov 3;21:307. doi: 10.1186/s12911-021-01659-8 (PMC8565006; doi:10.1186/s12911-021-01659-8)
Supplement: Supplementary file 4 — Additional file 4. Themes identified by the Reference Panel. [file 12911_2021_1659_MOESM4_ESM.pdf]

Additional File 4: Themes identified by the Reference Panel

The following 14 themes were developed from the feedback provided by the Reference panel:

1. Input from stakeholders at all stages
2. Sources of personal data and PERM data
3. Content of PERM
4. Interoperability
5. Workflow / Communication
6. Trust in the information
7. Safety and Security of data
8. Budget for all stages
9. User factors i.e. skills, education, background
10. Training
11. Design elements including Layout, Terminology, Use of Warnings/ Decision support
12. Existing electronic records/systems in place
13. Relevant Governance / Policies in existence
14. User access to PERM

Additional Themes added after brainstorming session before extraction began:

15. Usability
16. Issues affecting use of PERM
17. Awareness of others' role and SOP Changing roles of HCP in MedRec
18. Patient Tools
19. Patient’s rights
20. Technical Issues

These themes helped to develop the focus, given the time available, of what evidence we would look for in the included articles while still keeping an open mind for other evidence not identified by the panel.
