# Supplementary material for: Personal Electronic Records of Medications (PERMs) for medication reconciliation at care transitions: a rapid realist review
Source: BMC Med Inform Decis Mak. 2021 Nov 3;21:307. doi: 10.1186/s12911-021-01659-8 (PMC8565006; doi:10.1186/s12911-021-01659-8)
Supplement: Supplementary file 5 — Additional file 5. NVIVO codebook. [file 12911_2021_1659_MOESM5_ESM.pdf]

Additional File 5: NVivo Codebook

Nodes

Name	Description	Sources	References
Background	This is just me collecting some of phrases used in the introduction of the studies to summarise the background to the topic.	20	49
Citations of Interest	Any references that might yield additional data	15	29
Content of PERM	What information is available on the PERM	2	2
Barriers	Any stated perceived or actual barriers in relation to what content needs to be on the PERM or content that is on the PERM but has issues.	12	59
Existing Systems	What information was collected on existing PERM, if PERM systems already exist in the setting	17	34
New System	Any details in relation to the information to be collected or stored on the new system, if it differs from an existing system	18	87
Contexts		0	0
Design Contexts		25	167
Implementation contexts		20	88

Name	Description	Sources	References
Use Contexts		18	94
Data for Summary of Study Document	This is a way of gathering the information that I am populating the summary sheet for each of the studies.	25	166
Formal outcomes	A summary of the outcomes as stated by the researchers.	15	38
Frequency of use	by setting, by tool, by user	6	14
General Contexts	Any contexts not already specified	14	81
Awareness of others' role, SOP		7	11
Changing roles of HCP in MedRec		8	25
Evaluation process		3	7
Funding		5	7
Layout		6	33
Trust in information on PERM		7	13
Governance and oversight	Who manages the system overall, e.g. local, dept of health, national health service? Any reference to Local or National legislation, Policy or Guidelines	15	56
Mechanisms		0	0

Name	Description	Sources	References
Design Mechanisms	Confidence in information, Flexibility of software, attractive, intuitive, collaboration, respect, inclusion	4	10
Implementation mechanisms	Confidence in information, trusting (perception of accuracy), Flexibility of software, attractive, intuitive, collaboration, respect, inclusion, enabled, engaged, involved, satisfied, contented, valued, proud, determined, confident, opposed, ready, motivated, aware, understanding, skilled, incentivised, efficient, supported	11	26
Use mechanisms	Confidence in information, Flexibility of software, attractive, intuitive, collaboration, respect, inclusion	16	93
Patient focused tools	Any reference to consideration of patients in the design, implementation or use of PERM	13	50
Patient or person rights		11	26
Gaining patient consent	Any reference to the consideration of gaining patients consent and the scenarios around this topic, stakeholders' opinions on the issue etc	6	9
Realist Outcomes	Not the formal outcomes stated by the researchers, but the Realist outcomes, relating to how the context and mechanisms created these outcomes, unexpected outcomes, these may overlap with the formal outcomes.	0	0
Design outcomes	Relationship between stakeholders (Patients, Pharmacists, GPs, Hospital Staff),	4	7

Name	Description	Sources	References
Implementation outcomes	Confidence in information, trusting (perception of accuracy), Team effort, improved Workflow, improved Communication, Optimize human well-being and overall system performance	8	15
Use outcomes	Some examples given in the PROTOCOL: improved and efficient MedRec at CT, Improved information sharing, Perceived Potential Benefits, Increase efficiency, Reduced errors, Increased patient safety, Increased adherence to medications, Patients more aware of medications and reasons for use, Systems Engineering Initiative for Patient Safety (SEIPS), Considering impacts for all stakeholders in the ‘system’, patient confidence in the healthcare system, retention of workforce, staff morale and wellbeing	15	74
Research Question or Aim of study	As stated by the researchers	17	23
Hypothesis or Theories	Any statements regarding how the intervention or system was supposed to work- formal or informal	3	5
Safety and Security	Any references to issues that might affect safety of patients, data, staff etc.	15	53
Use of Warnings in PERM	Any reference to the use of warnings within the PERM, positive or negative	9	24
Setting	acute care, community care, nursing home/LTC, primary care, rehab etc	6	11
Study conclusions	As summarised by the researchers.	20	45
Training	Person (who will need it), Task (what do they need to be able to do), Work environment (where, equip etc) Resource (what is needed)	16	63

Name	Description	Sources	References
Types of PERM	Broad description of the function of the PERM i.e. dispensing, prescription etc	12	25
Usability	The broad topic of how the PERM is used, or not	8	24
Access	Who has access, where do they have access, when do they have access etc	9	21
Interoperability	Specific to different PERM systems, including references to how it is considered in design, implementation or use.	9	36
User Role	Medical: GP, Consultant, Specialist, Medical student, Physician Assistant Pharmacy: Pharmacist, Pharmacy technician. Nurse etc.	2	7
User role at design stage	Any details of who was involved at design stage, who provided feedback, at what stage they were involved, what types of input or changes were made based on their input/feedback. Any challenges, benefits etc	8	18
User role at implementation stage	Any details of who was involved at implementation, who provided feedback, at what stage they were involved, what types of input or changes were made based on their input/feedback. Any challenges, benefits etc	8	18
User role at use stage	Any details of who was involved in the use stage, who provided feedback, at what stage they were involved, what types of input or changes were made based on their input/feedback. Any challenges, benefits etc	14	68
Workflow or Communication	Any references to workflow in old or new systems, lack of workflow, breakdown of the workflow etc. Any references to how stakeholders communicate or lack of communication and the impact of this.	18	113

Sources and references from the HIQA policy documents relating to the ten theories for the Irish context.

NAME	DESCRIPTION	SOURCES	REFERENCES
Theory 1 Stakeholder Engagement	If users are given the opportunity to provide input, and both give and receive feedback at all stages of the introduction of a PERM system, they will feel engaged, be supportive, understand the challenges, be accepting and feel confident about the introduction of a PERM system.	7	33
Theory 2 Collaborative Design	If a PERM system is designed with user input, in relation to the display features and ease of use, with a balanced selection of Alerts or Warnings suitable to the setting, considers terminology issues, shows how complex issues will be dealt with early in the process and how user feedback has been taken on board, then users will feel heard and supported, thus fostering successful collaboration and acceptance of the PERM system.	8	85
Theory 3 Base PERM on existing tried and tested processes	If the content of a PERM system replicates Med Rec processes and forms that are already in existence in a setting, that have been shown to work well, then the PERM will feel familiar and consistent, users will feel confident using it and the PERM will become embedded more easily into normal work practices, allowing a smooth transition to PERM to improve Med Rec at care transitions.	8	74
Theory 4 Build Trust	If users are made aware of how others access and use the information on a PERM system, the sources of data that populates the PERM and how data is protected, their trust and confidence in the system will increase and they will comprehend how the system aims to improve patient safety and MedRec at care transitions.	5	29

Theory 5 Tailored Training	If training is provided to users that takes into account their existing knowledge of Med Rec at care transitions and their computer skills, and the training outlines the clear benefits, usefulness and usability of a PERM system, they will feel less anxious, be more engaged and confident in relation to the introduction of a PERM system in their setting.	8	55
Theory 6 Available and repeat training	If training on a PERM system is provided at implementation and continued at regular intervals to cater for new staff or those needing additional support, is available at times or in formats that suit all users, with the opportunity for users to give feedback and they are given time to become familiar with the system, then the users will feel supported and enabled to use the PERM system consistently thereby improving Med Rec at care transitions.	2	9
Theory 7 Resource Investment	The interoperability of a PERM system for Med Rec at care transitions increases the amount of data gathered, recorded and used. If organisations are made aware and acknowledge from the outset that this will impact on the workload of the users and understand the health economic value of a PERM system, they will be prepared for the additional resources required, which will improve access and organisation of data in a useful and useable format, facilitate more informed decision making, reduce errors, reduce	7	105
Theory 8 Positive impact of Legislation or Governance	If the introduction of a PERM system or standards for the MedRec process is supported by relevant legislation, governance or policies then organisational participation and engagement is increased impacting positively on individual users' engagement with the introduction of a PERM system to improve Med Rec at Care Transitions.	6	44
Theory 9 Patients as users of a PERM	If patients are provided with education in relation to the purpose and importance of adherence to their medications and are given training on the	3	11

	use of a PERM to support that, they will feel enable, empowered and organised in relation to playing a role in the maintenance of an accurate record of their medications, be more informed and have improved adherence to their medications impacting positively on their health.		
Theory 10 Interoperability<br><br>(Added after pilot)	If the PERM data sources are technically interoperable with the system, allowing integration of data from multiple sources then users will find the system aligns with the MedRec process flow and see the benefits, thereby increasing their use of PERM for MedRec at care transitions impacting positively on patient safety.	3	14
