# Supplementary material for: Personal Electronic Records of Medications (PERMs) for medication reconciliation at care transitions: a rapid realist review
Source: BMC Med Inform Decis Mak. 2021 Nov 3;21:307. doi: 10.1186/s12911-021-01659-8 (PMC8565006; doi:10.1186/s12911-021-01659-8)
Supplement: Supplementary file 6 — Additional file 6. Template for summary of study document. [file 12911_2021_1659_MOESM6_ESM.pdf]

Additional File 6: Template for summary of study document

Author	Article Title	Year	Type of Article
Country:	Ethics:		
Relevance (to topic):	Rigour (study design):	Richness (quality of data 0-4):	
Justification:	Justification:	Justification:	
Research Question			
Formal Outcomes			
Study Conclusions			
Any Theory/Concept outlined or inferred			
Software in use			
Setting – (Hospital, Nursing Home etc)			
Personnel involved			
Themes Identified			
Forward/Backward References			

Codes for Quality Assessment:

Relevance Rating: (0-3) 0= very weak, 1 = weak, 2 = good, 3 = very good

Rigour Rating: (0-3) 0 = very poor, 1= poor, 2 = good, 3 = very good

Richness Rating: (0-4) 0 = nothing of interest, not focused on D, I or Use, 1 = limited data of interest, likely to appear in other articles, 2 = limited data of interest, but quick to extract it and could add weight to findings, 3 = Some good quality data, 4 = Valuable data
