# Supplementary material for: Personal Electronic Records of Medications (PERMs) for medication reconciliation at care transitions: a rapid realist review
Source: BMC Med Inform Decis Mak. 2021 Nov 3;21:307. doi: 10.1186/s12911-021-01659-8 (PMC8565006; doi:10.1186/s12911-021-01659-8)
Supplement: Supplementary file 7 — Additional file 7. The supporting contexts, mechanisms and outcomes configurations. [file 12911_2021_1659_MOESM7_ESM.pdf]

Additional File 7: The context, mechanism and outcome configurations

Engage Stakeholders				
**If**	**Context**	**Mechanism**	**Outcome**	**Sources**
stakeholders including all user groups, at all stages of the design, implementation and use of a PERM system	Give input from both top down and bottom up,	they will feel engaged, be supportive, understand the challenges, accept, feel confident	Use of a PERM system for MedRec at care transitions, Improved inter-professional relationships	1, 2, 3, 4, 5, 9, 10,13, 14, 15, 16, 17, 18, 19
	Have opportunities to give and receive feedback			
	Facilitate role changes to allow work on development and implementation of PERM			
	Take account of users experience in their roles			
	Provide updated reports to users during implementation			
	Are provided with a supportive culture within the organisation			
	Evaluate at all stages, facilitated by moderators			
	Use a user-friendly evaluation feedback tool			

Inclusive Design				
**If**	**Contexts**	**Mechanism**	**Outcome**	**Sources**
a PERM system is	designed with user input	then users will feel heard and supported, thus fostering successful collaboration, acceptance and increased	use of the PERM system to complete MedRec at care transitions.	1, 2, 4, 6, 7, 8, 9, 11, 15,16, 17, 18, 19
	employs user-centered design & usability principles			
	Takes into account the general level of technology already in use			
	Has a balance of Alerts, Warnings, Range of Checks in the system			
	Considers display features			
	Considers layout for patients that is different to HCP			
	Keeps steps in the process to a minimum			
	Deals with Shorthand/ abbreviations			
	Provides detail info "on demand" to avoid clutter			
	Terminology: Medication information formatting.			
	Deal with the complex issues early in the process			
	Ensures users are happy with pilot versions			
	Works with real data at pilot stage			

PERMs complement existing good processes				
**If**	**Contexts**	**Mechanism**	**Outcome**	**Sources**
	Complements cognitive and workflow processes or forms already in existence	the PERM will feel familiar	PERM embedded	2, 6, 8, 9, 10,

the MedRec processes or PERM system	The existing MedRec processes are shown to work well	and consistent, users will feel confident using it	into work practices, a smooth transition, improve MedRec, users cognitive burden reduced	12, 15, 18
	The quality and performance of the software is acceptable			
	Caters for complex therapies/odd doses			
	Provides an opportunity to list reasons for medications /changes			
	Users are provided with time to get used to the new system			
	General level of IT already in use			
	Takes into consideration the computer skills of users			

**Build Trust**				
**If**	**Contexts**	**Mechanism**	**Outcome**	**Sources**
users	Are aware how they and others access and use the PERM system	their trust and confidence in the system design and use will increase	value and use it at care transitions, improve MedRec and patient safety, improved interprofessional relationships, improved awareness of others' roles	2, 4, 6, 7, 8, 8, 10, 12 13, 14, 15, 18, 19
	Understand the integrity of the PERM data sources, and the data protection controls			
	Trust the accuracy of the data			
	Understand the processes or roles of others			
	Are aware of how PERM Improves patient safety			
	Accept accountability for data entered			
	Are aware of others need for/ interest in data			

**Tailored Training**				
**If**	**Contexts**	**Mechanism**	**Outcome**	**Sources**
training is provided to users that	Considers their existing MedRec knowledge and skills	then feel less anxious and be more engaged and confident	Successful introduction of a PERM system, improved MedRec processes	1, 2, 3, 4, 6, 7, 8, 10, 12, 13, 14, 15, 16, 18, 19
	Considers their computer skills			
	Considers their role and responsibility in the MedRec process			
	Outlines the clear benefits, usefulness and usability of using a PERM system			
	Provides time to get used to the new system			
	Discussed any previous bad experiences using a PERM system			
	Provides one to one training if needed			
	Gives opportunity to provide feedback on training			

**Support and on-demand training**

If	Contexts	Mechanism	Outcome	Sources
users are	Provided with support and training on MedRec and the PERM system as needed	users will feel supported and enabled	use the PERM system consistently, improved MedRec at care transitions	4, 6, 7, 8, 10, 12, 13, 15, 16
	Provided with one to one training if needed			
	Provided with basic IT training if needed			
	Provided with support and training at times or in formats that suit all users			
	Provided with opportunities to give feedback on the training			
	New staff users are provided with support and training			

Interoperability				
**If**	**Contexts**	**Mechanism**	**Outcome**	**Sources**
the PERM system	Has interoperable data sources	Feelings of connectivity, alignment and efficiency	improves MedRec process flow, increased use of PERM	1, 2, 6, 11,12, 16, 19
	Allows integration of data from multiple sources			
	Allows automated data entry			
	Uses drug knowledge databases			

Resource investment				
**IF**	**Contexts**	**Mechanism**	**Outcome**	**Sources**
leaders/ management / organisations	Recognise the increased effort, volume and quality of data gathered	understand the need for additional resourcing	opportunities for risk identification, management and analysis, improved MedRec at care transitions and patient safety.	3, 8, 11, 12, 15, 18
	Value the opportunities the additional data provide			
	Provide sufficient funding from outset			
	Accurately cost the system			
	Can justifying the outlay			
	Recognise that improved MedRec increases workload			
	Recognise the need for additional human and technical resources			
	Recognises the need for continual on the ground support from IT			
	Consider funding for incentivisation			

Positive impact of Legislation or Governance				
**If**	**Contexts**	**Mechanism**	**Outcome**	**Sources**

the introduction of a PERM system or standards for the MedRec process	Are supported by relevant legislation, governance, or policies<br>Are considered in a National Strategy<br>Adapt to the adapting roles of the users	organisational participation and engagement	Increased user engagement with a PERM system, improved MedRec at care transitions.	1, 2, 3, 4, 6, 7, 8, 10, 11, 12, 13, 16, 18, 19

Patients as users of PERMs				
**If**	**Contexts**	**Mechanism**	**Outcome**	**Sources**
patients are supported to	Use technology and a PERM system<br>Understand their medical conditions<br>Understand the purpose of their medications<br>Record their medication use on a PERM system<br>Share their medication information using a PERM system	Feel enabled, empowered and organised	Maintain accurate medication records, be more informed, improved adherence to medications	1, 6, 7, 8, 9, 11, 13, 17, 19
