# Supplementary material for: Personal Electronic Records of Medications (PERMs) for medication reconciliation at care transitions: a rapid realist review
Source: BMC Med Inform Decis Mak. 2021 Nov 3;21:307. doi: 10.1186/s12911-021-01659-8 (PMC8565006; doi:10.1186/s12911-021-01659-8)
Supplement: Supplementary file 8 — Additional file 8. Introduction for expert and reference panels. [file 12911_2021_1659_MOESM8_ESM.pdf]

Additional File 8: Introduction for the Expert and Reference Panels
Review title.
What is it - about how, why, when and for whom Personal Electronic Records of Medications (PERM) are designed, implemented or used in practice at care transitions - that impacts on medical reconciliation? A Rapid Realist Review.

Definition of Terms for the purpose of this review:
Medication Reconciliation: Any process taken to compare one list of medications with another
Care Transition: Any movement between care settings or change in responsibility of care of a patient
PERM: Any electronic system used to record and store information regarding the medications (past or current) prescribed, dispensed or used by patients that may contribute to a record of their medication history.

Background
Medication reconciliation (MedRec) is a process to facilitate patient safety whenever patients transfer between care settings or providers. Lack of access to accurate information on patients' medicine use increases the risk of medication errors and poses a threat to patient safety.

Whilst evidence is emerging regarding the positive impact of Personal Electronic Records of Medications (PERM) implemented in research environments, very little information is available regarding how the design or implementation of a PERM system in the 'real world' impacts on the users' engagement with the technology and processes. We aimed to respond to this gap in knowledge by undertaking a rapid realist review (RRR) of the literature in relation to the introduction of a PERM system for MedRec at care transitions.

Why a rapid realist review?
Other researchers have examined the effectiveness of using a PERM system to improve MedRec at care transitions, we wanted to focus our research on developing a better understanding of what, how, why, when, where and for whom the design, implementation and use of a PERM system for medication reconciliation at care transitions is effective or not. Given the complex, multifaceted nature of strategies and interventions used to promote evidence-informed healthcare, and the current limited understanding of their mechanisms of action, the realist approach is particularly suited to the synthesis of evidence about complex implementation interventions. It is the theory of why and how the intervention was supposed to work which is the unit of analysis in a realist review, not if it worked.

More specifically, a realist review aims to identify what it is about interventions that generate change (i.e., the mechanisms) and under which circumstances the mechanisms are triggered (i.e., the contexts), which result in changes in the behaviour of the participants and/or implementers of the intervention (i.e., the outcome). These three elements, context, mechanism and outcome, are presented together as a statement or theory which attempts to describe what needs to happen for the intervention to work.

A rapid realist review is a more focused and accelerated version of a full realist review which aims to produce theories in a time-sensitive way and that is useful to a specific audience and/or emerging issues, while preserving the core elements of realist methodology. The methodology is guided by methodological guidance, publication standards and training materials for realist and meta-narrative reviews: Realist And Meta-narrative Evidence Syntheses: Evolving Standards (RAMESES) and training materials, which have been followed in this review.

A vital part of a RRR is input from people 'on the ground', providing local knowledge and context (the Reference Panel), and experts in the field from around the world (the Expert Panel) who ensure we reflect the most current thinking on the topic. For this RRR the Reference Panel is made up of key stakeholders, providing insight from community and hospital pharmacists, doctors, nurses, patients, safety science, informatics, human factors expertise, e-health, governance, policy, research and academia. The Expert Panel is made up of key researchers in the area, from America, Canada, Ireland, Sweden and the UK.

Review questions
We searched the literature to answer the following questions:

Q1. What are the contextual factors that have most impact (positive or negative) on the use of PERM for medication reconciliation at care transitions and for whom?
From a Realist perspective, examples of contexts relevant to this review include, but are not limited to, issues such as work environment, resources (i.e. investment, equipment, staffing, training) and governance, system issues such as interoperability, accuracy, reliability, security, user interface, user access, user workload, user's (computer) skills, sources of information, patient consent and stakeholders readiness to change.

Q2. What mechanisms are triggered in individuals and organisations using PERM that impact (positively or negatively) on the medication reconciliation process at care transitions?
From a Realist perspective, mechanisms relevant to this review are about users or organisations beliefs / feelings about PERM and related contexts (as listed above). Examples of mechanisms include, but are not limited to, being enabled, engaged, involved, trusting, satisfied, contented, valued, proud, determined, confident, opposed, ready, motivated, aware, understanding, skilled, incentivised, efficient.

Q3. What are the intended and unintended outcomes from the use of PERM in care transition?
The outcomes for this Realist Review will include anything that has impacted positively or negatively on the medication reconciliation process at care transitions. Examples of outcomes might include but are not limited to, Workflow, Communication, Relationship between stakeholders (Patients, Pharmacists, GPs, Hospital Staff), Safety, Efficiency, Errors, Adherence to medications, Patients awareness of medications and reasons for use.

The search of the literature resulted in a total of 656 articles, of which, after several stages screening, 52 articles were included in the review. These 52 articles were rated for "Richness" of data on a scale of 0-4. To date, those articles rated 4 (20) have had data extracted using NVIVO.

The extracted data allowed the development of ten theories in relation to what is it about how, why, when, where and for whom PERM are designed, implemented or used in practice at care transitions that impacts on medical reconciliation.

What we need from the Reference and Expert Panels
We need you, the reference and expert panels, to review the theories and provide some feedback using your own knowledge and personal experience in the field. You will be asked to indicate your thoughts on a scale of 1 -5 in relation to; how well you understand each theory, the relevance of the theory and how feasible you think it would be to apply the theory in practice. You will then be asked to comment and/or amend each theory; you may agree or disagree with them, suggest amendments you feel would improve them in relation to clarity and focus or simply make a general comment.

We will revise the theories based on your feedback and if any gaps in knowledge are identified we will search the remaining articles specifically for any data relating to the knowledge gaps.
