# Supplementary material for: Personal Electronic Records of Medications (PERMs) for medication reconciliation at care transitions: a rapid realist review
Source: BMC Med Inform Decis Mak. 2021 Nov 3;21:307. doi: 10.1186/s12911-021-01659-8 (PMC8565006; doi:10.1186/s12911-021-01659-8)
Supplement: Supplementary file 9 — Additional file 9. Feedback survey. [file 12911_2021_1659_MOESM9_ESM.pdf]

RRR of the use of PERM for Medication Reconciliation at Care Transitions

Please read the introductory document before completing this questionnaire. You will be asked to rank each of ten theories in relation to how well you understood it, how relevant you feel it is to the research topic and how feasible it would be to apply in the real world (practical). The ranking scale is 1-5 with 1 being the lowest and 5 being the highest. You will then be asked to add comments or suggestions on how to improve each theory.
Once you have ranked all ten theories there will be an opportunity to add further comments in relation to areas you feel are important but are not covered by the theories or any additional thoughts you have on the topic or process. We welcome and encourage your honest feedback.
The questionnaire should take approximately 30-45 minutes to complete. We are very keen to receive your comments so please do take some time on the free text boxes.

1. Engage Stakeholders

If users are given the opportunity to provide input, and both give and receive feedback at all stages of the introduction of a PERM system, they will feel engaged, be supportive and understand the challenges, they will then accept and feel confident about using a PERM system to complete MedRec at care transitions.

1. Please rank this theory

	1	2	3	4	5
Understood	○	○	○	○	○
Relevance	○	○	○	○	○
Practicality	○	○	○	○	○

2. Please add any comments or suggestions for improvements to this theory

2. Collaborative Design

If a PERM system is developed using user-centered design & usability principles then users will feel heard and supported, thus fostering successful collaboration, acceptance and increased use of the PERM system to complete MedRec at care transitions.

3. Please rank this theory

	1	2	3	4	5
Understood	○	○	○	○	○
Relevance	○	○	○	○	○
Practicality	○	○	○	○	○

4. Please add any comments or suggestions for improvements to this theory

3. Base the PERM on existing tried and tested processes

If the content of a PERM system replicates MedRec processes and forms that are already in existence in a setting and have been shown to work well, then the PERM will feel familiar and consistent, users will feel confident using it and the PERM will become embedded more easily into normal work practices, allowing a smooth transition to PERM to improve MedRec at care transitions.

5. Please rank this theory

	1	2	3	4	5
Understood	○	○	○	○	○
Relevance	○	○	○	○	○
Practicality	○	○	○	○	○

6. Please add any comments or suggestions for improvements to this theory

4. Build Trust

If users are made aware of how others access and use the information on a PERM system, the integrity of the sources of data that populate the PERM and the integrity of how data are protected, their trust and confidence in the system will increase, they will then comprehend how the system aims to work and be more likely to use it at care transitions to improve MedRec and patient safety.

7. Please rank this theory

	1	2	3	4	5
Understood	○	○	○	○	○
Relevance	○	○	○	○	○
Practicality	○	○	○	○	○

8. Please add any comments or suggestions for improvements to this theory

5. Tailored Training

If training is provided to users that takes into account their existing MedRec knowledge and skills, their computer skills and their role at care transitions, and the training outlines the clear benefits, usefulness and usability of a PERM system, users will then feel less anxious and be more engaged and confident in relation to the introduction of a PERM system in their setting.

9. Please rank this theory

	1	2	3	4	5
Understood	○	○	○	○	○
Relevance	○	○	○	○	○
Practicality	○	○	○	○	○

10. Please add any comments or suggestions for improvements to this theory

6. Available and repeat training

If training on a PERM system is provided at implementation and continued at regular intervals to cater for new staff or those needing additional support, at times or in formats that suit all users, with the opportunity for users to give feedback and they are given time to become familiar with the system, then the users will feel supported and enabled to use the PERM system consistently thereby improving MedRec at care transitions.

11. Please rank this theory

	1	2	3	4	5
Understood	○	○	○	○	○
Relevance	○	○	○	○	○
Practicality	○	○	○	○	○

12. Please add any comments or suggestions for improvements to this theory

7. Interoperability

If the PERM data sources are technically interoperable with the system, allowing integration of data from multiple sources then users will find the system aligns with or improves the MedRec process flow, thereby increasing their use of PERM for MedRec at care transitions impacting positively on patient safety.

13. Please rank this theory

	1	2	3	4	5
Understood	○	○	○	○	○
Relevance	○	○	○	○	○
Practicality	○	○	○	○	○

14. Please add any comments or suggestions for improvements to this theory

8. Resource investment

If the required time and resource intensity of introducing and maintaining a PERM system for MedRec at care transitions is recognised and understood early by organisations and they acknowledge from the outset that it will increase the amount of data gathered, recorded and used, increasing the users' workload, then organisations will be prepared and budget for the additional resources required.

15. Please rank this theory

	1	2	3	4	5
Understood	○	○	○	○	○
Relevance	○	○	○	○	○
Practicality	○	○	○	○	○

16. Please add any comments or suggestions for improvements to this theory

9. Positive impact of Legislation or Governance

If the introduction of a PERM system or standards for the MedRec process is supported by relevant legislation, governance or policies then organisational participation and engagement is increased impacting positively on individual users' engagement with the introduction of a PERM system to improve MedRec at care transitions.

17. Please rank this theory

	1	2	3	4	5
Understood	○	○	○	○	○
Relevance	○	○	○	○	○
Practicality	○	○	○	○	○

18. Please add any comments or suggestions for improvements to this theory

10. Patients as users of a PERM

If patients are supported to use PERM to understand and record their medication use and share their medication information, they will feel enabled, empowered and organised in helping to maintain an accurate medication record, be more informed and have improved likelihood of adherence to their medications.

19. Please rank this theory

	1	2	3	4	5
Understood	○	○	○	○	○
Relevance	○	○	○	○	○
Practicality	○	○	○	○	○

20. Please add any comments or suggestions for improvements to this theory

Your comments please

21. Please add your thoughts here in relation to any issues important to the design, implementation or use of PERM for Med Rec at care transitions that have not been addressed in the theories presented.
    We also welcome your comments in relation to the topic, the theories or the feedback process.

Did you need to consult the additional information document?

22. Question

- ○ Yes
- ○ No

23. If Yes, please indicate the number of any theory you needed to consult it for.

Thank you for providing your feedback

24. The feedback survey is anonymous, if you would like to share your identity with the researchers please enter your name below.

This content is neither created nor endorsed by Microsoft. The data you submit will be sent to the form owner.

Microsoft Forms
