# Supplementary material for: Personal Electronic Records of Medications (PERMs) for medication reconciliation at care transitions: a rapid realist review
Source: BMC Med Inform Decis Mak. 2021 Nov 3;21:307. doi: 10.1186/s12911-021-01659-8 (PMC8565006; doi:10.1186/s12911-021-01659-8)
Supplement: Supplementary file 10 — Additional file 10. List of included studies. [file 12911_2021_1659_MOESM10_ESM.docx]

**Additional File 10: List of Included Studies**

**Sources of Data Codes: Ax** Administration**, Dx** Dispensing, **EDS** Electronic Discharge Summary, **EHR** Electronic Health Record, **ETP** Electronic Transmission of Prescriptions, **HIE** Health Information Exchange system, **INS** Insurance Claim, **OCT** Over the Counter, **Pt** Patient, **Rx** Prescription.

**Relevance Rating**: (0-3) 0= very weak, 1 = weak, 2 = good, 3 = very good

**Rigour Rating**: (0-3) 0 = very poor, 1= poor, 2 = good, 3 = very good

**Richness Rating**: (0-4) 0 = nothing of interest, not focused on D, I or Use, 1 = limited data of interest, likely to appear in other articles, 2 = limited data of interest, but quick to extract it and could add weight to findings, 3 = Some good quality data, 4 = Valuable data

The richness ratings were all 4 at full text review stage and those presented below relate to revised ratings at the data extraction stage.

| **Author (Study Number)** | **Country** | **Title** | **Quality Assessment Ratings** | **Sources of PERM data** | **Type of system using PERM** |
| --- | --- | --- | --- | --- | --- |
| Ammenwerth et al  2014 **(1)** | Austria | A nationwide computerized patient medication history: Evaluation of the Austrian pilot project "e-Medikation" | Relevance 2  Rigour 2  Richness 3 | Rx, Dx | National System |
| Cadwallader et al  2013 (**2)** | USA | Design of a medication reconciliation application: facilitating clinician-focused decision making with data from multiple sources | Relevance 2  Rigour 2  Richness 3 | Rx, Dx, INS, | National System |
| Elliott et al  2020 **(3)** | Australia | Pharmacist-physician collaboration to improve the accuracy of medication information in electronic medical discharge summaries: Effectiveness and sustainability | Relevance 2  Rigour 3  Richness 4 | Rx, EDS, | Discharge Summary |
| Foged et al  2018 **(4)** | Denmark | Nurses' perspectives on how an e-message system supports cross-sectoral communication in relation to medication administration: A qualitative study | Relevance 1  Rigour 3  Richness 2 | Ax | E-messages to support Electronic lists |
| Hackl et al  2014 **(5)** | Austria | Crucial factors for the acceptance of a computerized national medication list: insights into findings from the evaluation of the Austrian e-Medikation pilot | Relevance 2  Rigour 2  Richness 3 | Rx, Dx | National System |
| Hammar et al  2014 **(6)** | Sweden | Implementation of a shared medication list: physicians' views on availability, accuracy and confidentiality | Relevance 2  Rigour 2  Richness 4 | Rx, Dx | National System |
| Kramer et al  2007 **(7)** | USA | Implementation of an electronic system for medication reconciliation | Relevance 3  Rigour 3  Richness 4 | Pt, Rx, EDS | Hospital system |
| Kusnadi  2012 **(8)** | UK | Moving from paper based to electronic hospital discharge summaries: a mixed methods investigation | Relevance 3  Rigour 3  Richness 3 | EDS drawn from hospital Ax and Rx data | Hospital system |
| Marien et al  2019 **(9)** | Belgium | A User-Centered design and usability testing of a web-based medication reconciliation application integrated in an eHealth network | Relevance 2  Rigour 2  Richness 3 | Medication list compiled by GP or HCP or pt | National System |
| Ng et al  2013 **(10)** | Australia | Medication reconciliation challenges at discharge from hospital using an electronic medication management system and electronic discharge summaries | Relevance 3  Rigour 2  Richness 4 | MoA, discharge Rx, EDS | Hospital system |
| Pandolfe et al  2016 **(11)** | USA | Medication Harmony: A Framework to Save Time, Improve Accuracy and Increase Patient Activation | Relevance 2  Rigour 1  Richness 3 | HIE drawing data from the patient and a variety of HCP | National System |
| Pellegrin et al  2018 **(12)** | USA | A Statewide Medication Management System: Health Information Exchange to Support Drug Therapy Optimization by Pharmacists across the Continuum of Care | Relevance 3  Rigour 3  Richness 4 | HIE drawing data hospital EMR | National System |
| Rangachari  2018 **(13)** | USA | Implementing a social knowledge networking (SKN) system to enable meaningful use of an EHR medication reconciliation system | Relevance 3  Rigour 2  Richness 4 | N/A | Hospital System |
| Rangachari et al  2019 **(14)** | USA | A Health System's Pilot Experience with Using Mobile Social Knowledge Networking (SKN) Technology to Enable Meaningful Use of EHR Medication Reconciliation Technology | Relevance 3  Rigour 2  Richness 4 | N/A | Hospital System |
| Salloum  2011 **(15)** | UK | The adoption of the national programme for information technology in the NHS: the case of Lorenzo | Relevance 3  Rigour 2  Richness 4 | Rx in EHR and ETP | National System |
| Sheehan et al  2018 **(16)** | USA | Helping Older Adults Improve Their Medication Experience (HOME) by Addressing Medication Regimen Complexity in Home Healthcare | Relevance 2  Rigour 3  Richness 3 | Medication list completed by home healthcare nurse and EHR | Patient EHR |
| Siek et al  2010 **(17)** | USA | Colorado Care Tablet: the design of an interoperable Personal Health Application to help older adults with multimorbidity manage their medications | Relevance 2  Rigour 2  Richness 3 | Patient built with Dx data available | Patient EHR |
| Smith  2006 **(18)** | UK | Exploring the effects of the electronic patient record on hospital pharmacy personnel using a case study approach | Relevance 2  Rigour 2  Richness 3 | EHR, Dx, Rx, Pt | Hospital and Pharmacy linked EHR |
| Tamblyn et al 2018 **(19)** | Canada | Improving patient safety and efficiency of medication reconciliation through the development and adoption of a computer-assisted tool with automated electronic integration of population-based community drug data: the RightRx project | Relevance 3  Rigour 3  Richness 4 | Rx, Dx, EHR | Hospital and Pharmacy linked system |

1. Ammenwerth E, Duftschmid G, Gall W, Hackl WO, Hoerbst A, Janzek-Hawlat S, et al. A nationwide computerized patient medication history: Evaluation of the Austrian pilot project "e-Medikation". International Journal of Medical Informatics. 2014;83(9):655-69.

2. Cadwallader J, Spry K, Morea J, Russ AL, Duke J, Weiner M. Design of a medication reconciliation application: facilitating clinician-focused decision making with data from multiple sources. Appl Clin Inform. 2013;4(1):110-25.

3. Elliott RA, Tan Y, Chan V, Richardson B, Tanner F, Dorevitch MI. Pharmacist-physician collaboration to improve the accuracy of medication information in electronic medical discharge summaries: Effectiveness and sustainability. Pharmacy. 2020;8(1).

4. Foged S, Nørholm V, Andersen O, Petersen HV. Nurses' perspectives on how an e-message system supports cross-sectoral communication in relation to medication administration: A qualitative study. J Clin Nurs. 2018;27(3-4):795-806.

5. Hackl WO, Hoerbst A, Duftschmid G, Gall W, Janzek-Hawlat S, Jung M, et al. Crucial factors for the acceptance of a computerized national medication list: insights into findings from the evaluation of the Austrian e-Medikation pilot. Appl Clin Inform. 2014;5(2):527-37.

6. Hammar T, Ekedahl A, Petersson G. Implementation of a shared medication list: physicians' views on availability, accuracy and confidentiality. Int J Clin Pharm. 2014;36(5):933-42.

7. Kramer JS, Hopkins PJ, Rosendale JC, Garrelts JC, Hale LS, Nester TM, et al. Implementation of an electronic system for medication reconciliation. American Journal of Health-System Pharmacy. 2007;64(4):404-22.

8. Kusnadi K. Moving from paper based to electronic hospital discharge summaries : a mixed methods investigation. [U - Thesis ; (Thesis (Ph D ))]. In press 2012.

9. Marien S, Legrand D, Ramdoyal R, Nsenga J, Ospina G, Ramon V, et al. A User-Centered design and usability testing of a web-based medication reconciliation application integrated in an eHealth network. Int J Med Inform. 2019;126:138-46.

10. Ng C, Welch SA, Luddington J, Bui D, Glasson E, Richardson KL. Medication reconciliation challenges at discharge from hospital using an electronic medication management system and electronic discharge summaries. Journal of Pharmacy Practice and Research. 2013;43(1):25-8.

11. Pandolfe F, Crotty BH, Safran C. Medication Harmony: A Framework to Save Time, Improve Accuracy and Increase Patient Activation. AMIA Annu Symp Proc. 2016;2016:1959-66.

12. Pellegrin K, Chan F, Pagoria N, Jolson-Oakes S, Uyeno R, Levin A. A Statewide Medication Management System: Health Information Exchange to Support Drug Therapy Optimization by Pharmacists across the Continuum of Care. Applied clinical informatics. 2018;9(1):1-10.

13. Rangachari P. Implementing a social knowledge networking (SKN) system to enable meaningful use of an EHR medication reconciliation system. Risk Management and Healthcare Policy. 2018;11:45-53.

14. Rangachari P, Dellsperger KC, Rethemeyer RK. A Health System's Pilot Experience with Using Mobile Social Knowledge Networking (SKN) Technology to Enable Meaningful Use of EHR Medication Reconciliation Technology. AMIA Annu Symp Proc. 2019;2019:745-54.

15. Salloum AA. The adoption of the national programme for information technology in the NHS: the case of Lorenzo. [U - Thesis ; (Thesis (Ph D ))]. In press 2011.

16. Sheehan OC, Kharrazi H, Carl KJ, Leff B, Wolff JL, Roth DL, et al. Helping Older Adults Improve Their Medication Experience (HOME) by Addressing Medication Regimen Complexity in Home Healthcare. Home healthcare now. 2018;36(1):10-9.

17. Siek KA, Ross SE, Khan DU, Haverhals LM, Cali SR, Meyers J. Colorado Care Tablet: the design of an interoperable Personal Health Application to help older adults with multimorbidity manage their medications. Journal of biomedical informatics. 2010;43(5 Suppl):S22-6.

18. Smith GM. Exploring the effects of the electronic patient record on hospital pharmacy personnel using a case study approach. [U - Thesis ; (Thesis (Ph D ))]. In press 2006.

19. Tamblyn R, Winslade N, Lee TC, Motulsky A, Meguerditchian A, Bustillo M, et al. Improving patient safety and efficiency of medication reconciliation through the development and adoption of a computer-assisted tool with automated electronic integration of population-based community drug data: the RightRx project. Journal of American Health Information Management Association 2018;25(5):482-95.
