# Supplementary material for: Personal Electronic Records of Medications (PERMs) for medication reconciliation at care transitions: a rapid realist review
Source: BMC Med Inform Decis Mak. 2021 Nov 3;21:307. doi: 10.1186/s12911-021-01659-8 (PMC8565006; doi:10.1186/s12911-021-01659-8)
Supplement: Supplementary file 11 — Additional file 11. Feedback responses. [file 12911_2021_1659_MOESM11_ESM.pdf]

Additional File 11: Feedback Responses from Panels

A total of 14 surveys were completed, one of these was a joint response from two experts working in the same organisation. This was a 88% response rate. The responses appear as they were presented, no spelling or typing errors were corrected.

Theory 1: Engage Stakeholders

If users are given the opportunity to provide input, and both give and receive feedback at all stages of the introduction of a PERM system, they will feel engaged, be supportive and understand the challenges, they will then accept and feel confident about using a PERM system to complete MedRec at care transitions.

Summary of Feedback for Theory 1:

The reference and expert panels understood and found this theory relevant. The panel members highlighted the need for all users, not just what they described as "first generation users", to be involved in evaluation and updates following implementation of a new PERM system. The establishment of a responsive feedback mechanism, such as a user group, that has the power to seek enhancements that improve front end usability, would facilitate this. The diversity of the users was also stressed, they must all be able to engage with the processes, practically this would be through representatives. It was also felt important to clarify that some stakeholders may not be users, but they also need to be involved in the process.

The term "introduction" in the theory, in relation to the process, was felt to be unclear, it was important to clarify that this included all stages of design, implementation and use of a PERM system. The theory was amended to take on board all of these suggestions.

In addition, the panellists acknowledged that if the PERM system is intended to serve multiple organisations, for example a nationwide system, the ability of this system to cater for the diverse range of users and settings exactly has limitations; as one panel member put it - *"I think we need PERMs that are good enough that providers will want to use them even if they haven't participated in the design/ build/ implementation"* **P11**.

Full text of Feedback

P1: Could be good to clarify what you mean by "users". Is it only health care personnel? You write "all stages of the introduction of a PERM" system. As I understand it you do not mean during development and design before introduction. To me it is important that users are included in all stages from the first steps in design process.

P2: This is a good pre,ise but is pnly applicable to the first generation of users who are there for the the introduction, UNLESS the PERM provider establishes a responsive feedback mechanism such as a user group that has the power to seek enhancements that improve front end usability. (Based on experience of TEAMS Med Rec at Tallaght, one of the obstacles was that teh ongoing governance arrangements were only weakly committed to by senior hospital management and there was a gap between stated intention to make improvements and actual alloacation of ICT and pharmacist staff resources to support it. Thst led to taerdiness with updates to the drug database and there was a sense of disappointment among pharmacists that the hospital did not truly value the system,)

P3: Management of expectations is a critical part of any engagement as is discussion/undersatnding of limitations etc {discussed under Building Trust}

P6: If engaging with stakeholders, essential to be in a position to act on feedback. Difficulty getting buy in for a process which may take more time therefore essential to be very clear about risks and harm with current processes {discussed under Building Trust}

P8: There is an extensive list of users and it may be difficult to engage with them all individually which may require engagement through representatives.

P9: Crucially important to learn from users and have involvement in the design of the system, not just the introduction. Not sure this is captured here? They'll be supportive if it meets their needs and fits into their work practices. So rather than being involved in the introduction of a system (that they may or may not want or need) and expecting them to thus be supportive of it, the system should be built around them and their needs. So modify to in the design of and introduction of the system... In practice, thinking of doctors in hospitals or primary care, nurses particularly in hospitals, there's a very large workforce and there isn't a great understanding of the problems with routine medication histories and how to do a proper med rec process instead. So even with a good PERM system, will it be used to complete a (flawed) med history, or to complete a proper high quality med rec process? ; the translation into using it properly to complete MedRec may be the most problematic part of it. Not sure if that comment fits here. With pharmacists and technicians, I would be more confident that there's a good understanding of how to do good quality med rec so wouldn't have the same reservations.

P11: To the extent that a PERM may be a multi-institutional electronic application, there may be limited ability to continually rework it to respond the needs of new users. This theory seems good in concept, but somewhat difficult to apply across multiple institutions and a user base that is continually changing, even if only slowly. I think we need PERMs that are good enough that providers will want to use them even if they haven't participated in the design/build/implementation.

P12: Agree with this in relation to adapting a particpatory/co-design approach. Alot hinges on obtanining the participation of a range of stakeholders (i.e. mix - different relevant roles) and making use of the feedback provided

P13: Definitely think this is crucial step. Would suggest introduction of 'development' along with introduction and amending wording slightly as follows: If users are given the opportunity to provide input, and both give and receive feedback at all stages of the development and introduction of a PERM system, they are more likely to feel engaged, understand the challenges and be supportive of the need for change. They will then be more likely to accept and feel confident about using a PERM system to complete MedRec at care transitions.

P14: No comment recorded

Theory 2: Inclusive Design

If a PERM system is developed using user-centered design & usability principles then users will feel heard and supported, thus fostering successful collaboration, acceptance and increased use of the PERM system to complete MedRec at care transitions.

Summary of Feedback for Theory 2:

The panels' comments about this theory were mostly to endorse it. Only one issue was highlighted by an expert in human factors and safety science, who pointed out that even when the system reflects user friendly design principles it does not guarantee that the concepts for the system will be

right - “*User centred design is not necessarily a collaborative process - for example user testing does not mean 'designing with' - it is really about evaluation and 'designing for'*” **P12**. The theory needed to make it clear that user input will also be taken on board.

Full text of Feedback

P1: Good. I agree.

P3: Management of expectations is critical to this - there will be a journey to travel that needs to be understood. Achieving everything in one go is unlikely and means that delays will be inevitable unless a shared journey is understood with an iterative process

P8: There is an extensive list of users and it may be difficult to engage with them all individually which may require engagement through representatives.

P9: Great. It's involvement in informing the design that is crucial. This one is perfect.

P12: User centred design is not necessarily collaborative process - for example user testing does not mean 'designing with' - it is really about evalulation and 'designing for'. Applying princples does not guarantee that the concepts for the system will be right - it just means that the system will reflects user friendly design principles

P13: Perhaps something could be included to say that this approach is more likely to lead to a system that addresses the problem in a user friendly way. However I think it is good as is, and may be better to be concise!

P14: My slight hesitation around practicality relates to the time consuming nature that may be tricky when time is an issue in getting a system out - also reaching consensus across viewpoints and how this is managed

Theory 3: The PERM complements existing good processes

If the content of a PERM system replicates MedRec processes and forms that are already in existence in a setting and have been shown to work well, then the PERM will feel familiar and consistent, users will feel confident using it and the PERM will become embedded more easily into normal work practices, allowing a smooth transition to PERM to improve MedRec at care transitions

Summary of Feedback for Theory

The expert and reference panels agreed with the basis of this theory but almost unanimously stressed that the PERM system should not simply duplicate paper processes; the opportunity to improve on existing systems using socio-technical systems design methods that consider human, social and organisational factors, as well as technical factors and the wider design, implementation and use elements, should not be missed.

As with Theory 1, the feasibility challenge of agreeing on a MedRec process, and ultimately a PERM system to support it, that would work in diverse settings or nationally was highlighted.

Full text of Feedback

P1: Partly agree.

P2: Evolution is always better than revolution,. Even better id the evolution alows staff to retain familiarity while making the actualk work easier, for example by automating things that previosuly were manual, or that faciliates easier data input.

P3: Except that the adoption of technology should be part of a transformative approach and not a duplication of paper processes - this suggests the latter which is inappropriate

P4: This is true, but the MedRec processes/forms may vary amount stakeholders/sites and there would need to be a consistent agreed standard process & documentation for this to work nationally.

P6: This would be really helpful to users and help to make it seem intuitive if familiar with existing processes. May be problematic if differences in current documentation and processes between different sites

P7: This can be complicated as MedRec process is different depending on professional doing it and particulars of the setting.

P8: It is not always feasible or correct to simply replicate business processes as there are socio-technical considerations involved. There are gaps and challenges to introducing any health IT system when paper systems already exist. There can be opportunities for improvements in processes rather than replicating existing performas. Significant training is also required even if PERM attempts to replicate paper based processes.

P9: It might. But you might also be limiting the benefits of the PERM by trying to mimic existing processes etc. Could really limit the potential here. Even if it mimics an existing system completely, it's still transitioning to an electronic format, so the processes and interface is changing a lot. So yes, keep familiar terms and fields and certainly refer to what works well with existing systems but I think this would very much limit the potential of a new system.

P11: because processes and forms vary across organizations (and even within organizations), it may be difficult to design a PERM to replicate/reinforce these processes/forms

P12: Yes, this does make senese, but you also want to improve/transform the process. Simply digitizing a process is not enough

P 13: While this can be a tempting approach, there is a common trap that can be fallen into, whereby existing systems or processes are simply digitised, instead of looking for the most digitally powerful/effective way of completing a process, with further enhancements, opportunity to capture different/additional data that is structured and usable, incorporate clinical decision support and so on. I would suggest a more successful approach might to take the best of existing processes and forms, and look to blend these with the power of a digital solution and associated functionality. It can be even more effective to show users how new systems are going to enhance/enable their work and the demonstrate the benefits these new systems can bring to patient care/safety.

P14: No comment recorded

Theory 4: Build Trust

If users are made aware of how others access and use the information on a PERM system, the integrity of the sources of data that populate the PERM and the integrity of how data are protected, their trust and confidence in the system will increase, they will then comprehend how the system aims to work and be more likely to use it at care transitions to improve MedRec and patient safety.

Summary of Feedback for Theory

There was general acceptance that awareness of the integrity of the sources of medication data and data protection enhanced trust in the system. This partly involved an understanding of the authenticity of each data source, which may not itself be an accurate representation of the patient's medication use or exposure, but rather helps to build a more valid picture of it. The notion of the importance of how others use and access the information was less clear to them. There was substantial evidence in the articles to support this element but perhaps it was poorly described in the provisional theory, and therefore it was amended to clarify this.

Full text of Feedback

P2: This is often under appreciated because indivudal players may seer only their own part od the process. In this sense a good way to oversee any process like this is to have a formal Lateral Relation (see Jay Galbraith, orghanisations as information processors and Lateral Relations). Giving evryone full oversight enhances the sense of teamwhork and mutuality.

P8: We find this theory difficult to understand on the first attempt of reading it and it would benefit to be broken up or reworded differently.

P9: I am not sure that this "how others access and use the information on a PERM system" would make a difference. Integrity of data sources and protection, yes. Will build trust, confidence, comprehension and more likely to use, yes. Importantly they will understand the strengths limitations of the data and use it appropriately. This is a big concern with a PERM - even the best system still requires a med rec process, requires validation (with the patient), and if this isn't understood or done a PERM might just be used as the medication history, with resultant problems. Really important to capture that in any of the theories. I think this is an element that would be necessary, but I don't think it stands alone as a theory of how to get a PERM to be used well for Med Rec.

P11: PERMs will always have some erroneous data. The key is allowing users to understand where the data comes from (organization, date, user who obtained, how obtained (e.g. from pt, caregiver, pharmacy, whether the user felt the information to be complete or whether they knew some detail was unobtainable at that time) so they can know how to apply it.

P12: Yes, this is important - issues around data protection and data security - particularly for health information are now part of the public discourse. If the protections are explained to end useres, then I think this increases trust and acceptability. However, this does not mean that it will make the system more user friendly or appropriate for the task - this still requires participatory/stakeholder engagement. So, building trust around data use/storgage/access is just one factor to be addressed in relation to motivating adoption etc

P 13: I am clear on what the intent is here, but would suggest slight amendment to wording to clarify this - I'm not sure that them comprehending 'how the system aims to work' is the outcome. Suggested amendment below: If users are made aware of how they and others access and use the information on a PERM system, the integrity of the sources of data that populate the PERM and the controls in relation to how data are protected, their trust and confidence in the system will increase. This confidence and trust in the system and how it has been developed will make them more likely to use it at care transitions to improve MedRec and patient safety.

P14: No comment recorded

Theory 5: Tailored Training

If training is provided to users that takes into account their existing MedRec knowledge and skills, their computer skills and their role at care transitions, and the training outlines the clear benefits, usefulness and usability of a PERM system, users will then feel less anxious and be more engaged and confident in relation to the introduction of a PERM system in their setting.

Summary of Feedback for Theory

The reference and expert panels had extreme views on this theory; while most agreed it was an important element, others stated it was not relevant. Those who felt it was not relevant felt that if the system was well designed it should be intuitive and training should not be required. The cost and time to undertake training were also identified as issues; the literature suggests that the solution to this is to ensure that users only attended the training they need, in a format that suits them. Some way of assessing they have the training they need could be built into the system. This is what we intended when using the term “tailored” training.

Most panellists felt that easily accessible training should be available to users if and when needed – “The User Interface should be intuitive and supporting training videos, even by specific task(s), should be used to allow the users to quickly find and use to aid them” **P4**.

Full text of Feedback

P2: My concern ehr is the practicality of TAILORING training, There are so many competing demands on time for NCHDs and pharmacists and much of training has moved online, Perhaps this could have a tailored design, but that would add to the cost. For me, the improtant thing is to make a system sufficiently intuitive that pharmacists can learn it from colleagues and this could then be assessed by an expert user to make sure they had maximised their utilisation of the system. (This may qualify as tailoredf training but I see it as tailored assessment)

P4: It's not that relevant and would may require significant overhead to tailor training. The User Interface should be intuitive and supporting training videos, even by specific task(s), should be used to allow the users to quickly find and use to aid them.

P6: Tailored training would be great as likely wide variation in different users experience. Need to avoid overloading people with training about aspects they are already familiar with

P7: Bespoke training platform for different users at different care interfaces is essential to embed.

P8: Different levels of training may be required and individuals may require more support than others particularly in early implementation.

P9: Yes, this is important and absolutely ideally, this would happen. It can also act as a barrier - time to train, how it's delivered, when - so does it stop people getting access to a useful system? I am thinking particularly of medics who just don't get training time and move rapidly between workplaces. Maybe the first time you open it up (or come to a point in the process where you should), you get a few screens to explain it (you click Got it for each). So the most streamlined, straightforward training possible. Making the PERM system intuitive, easily used, with obvious benefits, would be better. I.e. we don't get training on using our iPhone but we have no problem. Training has very limited benefits when it comes to work processes. So make the system easy, obvious, design it into the processes. If training is needed it would be more focussed on addressing gaps in understanding and practice with delivering quality MedRec, withthe PERM system

mentioned in that regard. If the PERM system requires anything other than the most minimal training, it's not designed well and won't be used (as intended or at all, by many users).

P11: again this may be difficult with a diverse user base within and across organizations and over time. PERM designers may consider allowing for some different training/functionality based on these factors.

P12: Yes, this is obviously important - but again, only one factor which will impact on use and adoption. Have to consider co-design, clear communication about how data is used/protected, training, enabling easy access to systems etc

P 13: It is not always feasible or correct to simply replicate business processes as there are socio-technical considerations involved. There are gaps and challenges to introducing any health IT system when paper systems already exist. There can be opportunities for improvements in processes rather than replicating existing performas. Significant training is also required even if PERM attempts to replicate paper based processes.

P14: No comment recorded

Theory 6: Support and on-demand training

If training on a PERM system is provided at implementation and continued at regular intervals to cater for new staff or those needing additional support, is available at times or in formats that suit all users, with the opportunity for users to give feedback and they are given time to become familiar with the system, then the users will feel supported and enabled to use the PERM system consistently thereby improving Med Rec at care transitions.

Summary of Feedback for Theory

The panels had similar reactions to this theory as to Theory 5, the average rating was the lowest for relevance and second lowest for practicality of all the theories. Despite the low scores, the panellists could see some benefits to repeat training for users who needed it, but it must be balanced with the need for training in other areas. None of the panellists consider the issue of new staff or staff turnover which may account for the low scores for relevance and practicality. A few acknowledged that feedback was useful but saw it as a way of getting feedback on the system, which was not the focus of this theory. This theory was simplified and improved clarity.

Full text of Feedback

P4: Again, while initial training may be given, as per previous answer, training should be delivered via online videos that are available to users on demand and quickly allow to get up to speed on a specific process using the PERM. Feedback could also be provided electronically. This is more effective as it allows users to train/learn at a time convenient to them; with significantly less cost.

P6: Ideally the PERM should be as intuitive and user friendly as possible to minimise training needs. Training important to ensure people understand importance of the work.

P9: See previous comments. If the PERM system requires this level of training, it's not going to be used by some or all users, and it's not going to be used properly. If the training is about MedRec more generally, with the PERM system included, ok. But it shouldn't need to be repeated by the same person. Exception is staff who need or want more support (as above), should be help for them but not sure I'd even call it training, more user support.

P11: Of course users will do better will more available and repeat training, but this will need to be balanced with other training needs and resources

P12: Yes, this is important - once off training is not enough. I think it is key to get feedback about the system too - as you mention, this can be obtained in part through training. But it is important that the feedback is acted on - not a tick-box exercise. Training can be an opportunity to gather overlooked requirements/usability issues - once this does not hamper confidence in the system

P13: Understand the intend here, but think it probably needs a review in terms of readability. Perhaps breaking into 2/3 sentences.

P14: No comment recorded

Theory 7: Interoperability

If the PERM data sources are technically interoperable with the system, allowing integration of data from multiple sources then users will find the system aligns with the MedRec process flow and see the benefits, thereby increasing their use of PERM for MedRec at care transitions impacting positively on patient safety.

Summary of Feedback for Theory

This theory was well accepted by the panels but acknowledged as challenging to achieve. The issues identified by the respondents were concerns about the quality of the data from the source systems and semantic interoperability. A number of panellists commented that interoperability was only of use if the system was well designed; this is dependent on the contexts and mechanisms identified in some of the other theories – *"In practice, this could be very challenging to do well - designing the integration of data with inbuilt intelligent processing so there isn't a lot of duplication and annoying rubbish that needs to be cleaned up by the user. However, if it can be done well it would be great"* **P9**.

Full text of Feedback

P1: This is important but difficult. This is kind of what Sweden is aiming at but it is difficult and I believe you need the other parts from the other theories as well together with this to succeed. You need good working processes, routines and clear role/responsibility for each user.

P2: This is especially important as an enabler when there is access to patient records from the POCRS database, for example, or from any new clud-based Summary Care Record that includes a medication list.

P4: The data may be more complete and accurate, but the process around it will not necessarily just improve because of this. As the tool(s) improve, the processes should be reviewed & updated to make better use of the information; impacting positively on med safety.

P8: Some of the bigger issues regarding interoperability is the quality of the data from source systems, ability to update correct information from source systems, and the semantic interoperability is important in terms of coding information which may vary hugely across systems or not available. There may be delays to information getting into source systems which impacts the PERM for MedRec.

P9: This would be great, make it simpler to use. In practice, this could be very challenging to do well - designing the integration of data with inbuilt intelligent processing so there isn't a lot of duplication

and annoying rubbish that needs to be cleaned up by the user. However, if it can be done well it would be great.

P11: this is most relevant for patients who get care across multiple organizations

P12: Absolutely agree with this - I think this is likely a key success factor in adoption. One system that integrates everything is key to simplicity and will motivate adoption and acceptability. The system still has to be well designed - so if it integrates everything but is very confusing/poorly designed, this is going to act as a barrier.

P13: This is one where practicality will be the biggest challenge - old and creaking systems don't like interoperability! However, definitely needs to stay in, and is something that needs to always be worked towards and incorporated into design and development of any new system.

P14: No comment recorded

Theory 8 Resource investment

If the required time and resource intensity of introducing and maintaining a PERM system for MedRec at care transitions is recognised and understood early by organisations and they acknowledge from the outset that it will increase the amount of data gathered, recorded and used, increasing the users' workload, then organisations will be prepared and budget for the additional resources required.

Summary of Feedback for Theory

Although this theory was fairly well understood and felt relevant by the panellists, the feasibility of putting this theory into practice was scored the lowest of all of the theories.

A number of the panellists related poor personal experiences regarding the budgeting of such projects. The importance of the leaders/ management / budget holders understanding that improving MedRec does not only involve resourcing a PERM system to support it, but the potential benefits of sourcing improved data requires additional users to use this additional data, was highlighted

Full text of Feedback

P1: In this theory you do not include "improve MedRec at care transitions" in the end. If course time an resources are needed to make this happen and I agree it is relevant that the organizations understand this early. However, this is not in itself a solution if they are putting time and money on something going in the wrong direction.

P2: This is true but I scored it low on practicaluty based on]experience of how the Irish public health service funds developments, which is usually based on hagglinmg rather than on sensible, costed work plans. In practice the latter shpould be done. But the reality is otherwise in most cases. I have two examples, one national and one local: NATIONAL In 2017 acting as an advisor to HSE I did an exercise with senior HSE people showing a need for 292 WTE staff at senior pharmacist level to do med rec in acute hospitals. That was in 2017. The process engineer I worked with understood what was needed and supported it based on outr work,. But her reverted to me and told me that the only way the HSE Executive Board would agree it, was if it were phased into three phases over 3=6 years. In the end, the plan was phased but even that was not formally approved. However, the numbers of senior pharmacists increased by 41, 28 and 37 in each of the next three years giving a total of 108 additional staff,. And there was no explicit requirement that these staff be dep;loyed to Med Rexc,

or that Med Rec work be audited. LOCAL: In Tallaght we published our PACT paper in 2014 and with consultant leadership supporting us, we put forward internal business cases to TUH management to add 8 senior pharmacists for PACT form of Med Rec. We made no progress with this because the management said they had no funding AND there was an embargo on hospitals invcreasing their employment control numbers, so the ponly way we could get 8 staff is if other departments were reduced by 8 staff. So we had the frustrration of everyone saying they would love to help but.... Eventually we made a breakthrough by luck. The Sec Gen of hSE was Tony O'Brien and he liked to tweet. On Twitter her said he wanterd to support HSE staff with ideas for new watys of working. I tweeted him that I had been fighting to get permission to use new ways of working but that the only obstacle was mamangement and the HSE system,. That led to an email discussion and he got me a chance to pur a business case to the National Director of Acute Hospitals. We gor the needed funding to add eight staff and did so over the following 3 years. It was a difficult task because of the need to train these staff and get them to the level needed. Not for med rec but for PACT. And as time went on, my own management team comprpomised the fidelity of our intervention by passing up on one senior posts to relieve a pressure on the educationl and training side. So there was a failure of constancy of purpose within our own leadership.

P6: Challenging - need to focus on ensuring key decision makers really understand the implications of this work and buy in to it.

P8: Hard to generalise at both an organisational and individual user level. Organisations may have different resource capabilities to offer a MedRec solution at care transitions.

P9: If "increasing the users' workload" wasn't in this, I would understand and agree with this. There is a workload for development, monitoring, upkeep, data analysis etc that is considerable and often it's pharmacy and IT that need to provide this. But I don't see that this would increase the users' workload. The system should decrease the users' workload; it should help them do a process in a more streamlined way. So I agree with the rest of the statement, but think it's that the organisation needs to recognise and support the resources needed to develop, deliver and further develop the system and keep it up to date and responsive to issues arising. This should happen, I'm being honest with the practicality score, haven't met an organisation yet that has resourced this properly, particularly the ongoing maintenance and changes.

P11: some of the difficulties are that even though MedRec has strong face validity, there isn't great evidence to show that it impacts patient-relevant outcomes, especially in real-world settings. also, it's possible to go to extremes to do it nearly perfectly, as opposed to just sufficiently. but in the context of not having strong evidence, it's sometimes difficult to know how much to invest in it.

P12: I think organsiations tend to introduce tech with the assumption that it will reduce person resources required - i.e. build process/task efficiencies. And obviously to improve safey - the case around safety is well established for med reconciliation/PERM. Ideally systems should reduce effort and not increase effort - and machine intelligence should be used for this purpose. But the inital PERM tech may not be that sophisticated - so it may end up increasing task times. I think a key thing is that the time requirement for using this tech does not worsen the patient experience and impact on person centred care. From this perspective, org should identify if more resources are required so patient experience is not compromised - you don't want to pit patient experience and patient safety against each other. So I suppose I am saying that yes, org need to be prepared, but not simply look at this from a resource perspective - consider the impact on both staff and patients.

P13: I definitely agree resource investment is crucial and this needs to be recognised by senior management/whomever holds the purse strings. However I think the wording is a little unclear as is currently. I think rather than organisations undersetanding it, it needs to be clear that it is those in leadership roles. Also, while the new data being gathered may need to new outputs, I wouldn't agree that users' workload is necessarily going to increase - the intention surely would be that efficiencies would be delivered by introducing a technology solution, particularly if replacing paper-based systems. Suggested amendment below (it's too long, but there might be some bits that are useful): The time and resource required to introduce and maintain a PERM system for MedRec at care transitions must be recognised and understood early by leaders/management, and budgeted for appropriately. In addition, sufficient budget must be made available for training and implementation phases. They must also understand that volume and quality of data gathered will be increased, providing opportunities for new insights and risk identification, management and analysis - however, using this data may require new expertise or roles to be introduced. Expected efficiencies/time savings for users from introducing a new system should be calculated (e.g. using time in motion exercises) and highlighted as an expected positive output.

P14: No comment recorded

Theory 9 Positive impact of Legislation or Governance

If the introduction of a PERM system or standards for the MedRec process is supported by relevant legislation, governance or policies then organisational participation and engagement is increased impacting positively on individual users' engagement with the introduction of a PERM system to improve Med Rec at Care Transitions.

Summary of Feedback for Theory

This theory was well understood and felt relevant by all panellists - *"This makes everything an explicit commitment and removes all assumptions"* **P2**.

The practicality rating was lower. Of the four panellists scoring this theory 3 or lower, two panellists provided some comments to explain their score. They pointed out that regardless of organisational engagement, the PERM system still needed to be usable and fit for purpose as identified in earlier theories in order for the individual users to engage with the PERM system.

An interesting point made by one of the panellists related to the potential role of a positive organisational culture to improve engagement - *"I think a more powerful but harder to achieve ambition would be that culturally, it is recognised by the organisation that it is the right thing to do, and legislation, governance and policies are secondary to this (while still being important)."* **P13**.

Organisational culture was referred to in a small number of the included articles, but only one identified the lack of a positive organisational culture in relation to teamwork and open communications between all levels in the organisation as impacting on the failure to introduce a PERM system (27). There was insufficient evidence on which to develop a theory in relation to organisational culture being a context or mechanism to improve engagement in the process, but it seems likely that it would impact on the outcomes in a similar way to legislation or governance.

Full text of Feedback

P1: This is really important. In Sweden we have a new legislation to implement a nationally shared medication list and many actors are working together with this. Still, it is very complicated and there is a risk we will not reach the desired outcome.

P2: For mw this is vital to support staff in theior practice, especially where the new practice is giving them increased levels of responsibility. I think we have done this well in TUH by having Hospital BVoard apoprioval for PACT, as well as explicit approval for our way of working from the State Claims Agency and from the hospital Mecdical Director. The most important artefact is a Service Lebel Agreement that each consultant signs off in order to receive the clinical service,. This makes everything an explicit commitment and removes all assumptions.

P6: May be more powerful although need to ensure any additional requirements are feasible in practice at all times if they become legal requirements.

P9: It shouldn't breach any legislation or regulations, so yes if needed there should be adjustments to them, but I don't see how individual users would engage more or less as a result. Even national or organisational policies, they might drive it being adopted by organisations, but users will use it if it helps them and works for them and won't (or won't use it properly) if it's not easy, workable, helpful.

P11: in general, i believe the biggest obstacles to health information exchange are lack of a business case and exposure to liability for participating organizations

P12: Yes, I have seen this in aviation - where regulator demands certain types of tech/safety protections enabed by tech, and then good systems are introduced. But I think this is just one factor - and other things like participatory design/stakeholder involvement, implementation evaluation, training, data protection are all important too

P13: This is definitely true - but reckon it might be a tricky one to get right. If the motivation is meeting an audit/legislative tickbox, then the danger is all efforts will be driven towards hitting the milestone, and then attention is taken away. I think a more powerful but harder to achieve ambition would be that culturally, it is recognised by the organisation that it is the right thing to do, and legislation, governance and policies are secondary to this (while still being important).

P14: No comment recorded

Theory 10 Patients as users of a PERM

If patients are supported to use PERM to understand and record their medication use and share their medication information, they will feel enabled, empowered and organised in helping to maintain an accurate medication record, be more informed and have improved likelihood of adherence to their medications.

Summary of Feedback for Theory

This theory was well received by the panels, with several stating that a theory relating to patients' involvement was essential in this process. The benefits identified by the panellists were those stated in the theory.

Issues that impacted on the practicality of patients using PERM identified by the panellists related to their ability to maintain or engage with a PERM, either due to their cognitive abilities, technology skills, level of support available to them or the design of the PERM and how intuitive and easy it is to use.

Full text of Feedback

P1: This is also very important and I hope we will see much more of this from now on. In my research we try to look from all perspectives, both health care professionals, pharmacists and patients. But again, this in itself will not solve things. Several things are needed.

P2: I see this as becoming increasinlgy rlevant as patients move more and more to using Apps to manage their own meedication use, including documeting their self administration. That wpould giive is end to end control where the patient feels an actice participant in the therapy rather than a passive follower of instructions. However this type of approach does not work for all patients - people with impaired cognition, for example.

P6: I think the need for support is important to increase the number of people who increase involvement and not just continue with the same proportion who are already engaged. Not sure what this support would look like though

P8: Carers need to be included. Not all patients will have the capacity or ability to maintain a PERM.

P9: Some patients. Some patients would definitely fit this theory. Some patients will not, would find it a burden/impossible. Many patients wouldn't engage with it, lack of interest. So yes, some patients will feel enabled etc. The practicality would be higher or lower depending on how intuitive and easy to use it is - if the patient needs a lot of support, that will limit this, both the patient and healthcare professional won't have the time and willingness to do this.

P12: Yes, agree about patient enablement. Fostering/enabling self management is key. And end users need to be supported with this - taken on some form of training journey. I agree that if you explain the benefits to end users that this helps. In other areas of research (for example, assisted living), it has been clearly demonstrated that if you explain the benefits - paying attention to what might intrisically motivate a stakeholder/end user, you get more buy-in.

P13: I do think it is important to always consider the patient - it can just be difficult in reality to involve them in what can be a complex process. I would certainly think there needs to be a patient related theory however.

P14: No comment recorded

General Comments

P1: Very good and interesting and extremely relevant for my field of research. In many of the theories you write "users". If can be good to clarify what you mean by users. In your theories you describe several really relevant aspects. What I believe from my research is that a combination of several of these are needed. One thing that I didn't really find in any of the theories (I may have missed it) was the importance of a really good in depth understanding about medication management and the pharmacinformatics. What I mean here is that you need the full picture of what kind of information all of the actors need and when, which systems they are using, how they work etc. Perhaps this can be a part in the user design process. I also want to highlight the need for research and evaluation related to the implementation and use to be able to identify unintended effects and problems to improve the system.

P2: A public awareness campaign of the real dangers of medication misadventure at transitions of care is needed.

P3: This is a technically challenging area that is going to require an iterative approach to support both developments and delivery. A shared vision and a roadmap to get to the end goal will be needed if this is to be delivered - without this there is the danger that nothing will happen for a

considerable time when the reality is the starting to expose the information required, even in a non-interop manner, will still deliver significant benefit.

P9: Back to an earlier comment, MUST not depend on the PERM for med rec, i.e. must only use it as an information source while doing the proper med rec process. I.e. verify with the patient (or other reliable source if patient not able to verify). So introducing a PERM without good med rec practice may lead to more error not less (there are a few publications on this). Must be able to easily update it and it must be clear when it was last updated and verified. We have found that health care professionals have a very deep mistrust of patient accounts of their medication history but consider the GP or pharmacy record to be the true list. Whereas with med rec, we say that what the patient is actually taking is the true list and then you look at if anything is missing etc. This is a big issue to unpack for a PERM. If the data feeding into it is from prescribing, then the patient may not be taking it, may not take it in the same way as the prescription etc. If it's from dispensing, the patient has at least received it, but again may not be taking it as directed. If it's from the patient and they're filling it in themselves, it may have limitations. If it's a combination of these, there's a lot of intelligence required within the system to keep on top of all of that. And so really it has to accommodate the validation of it all when a proper, quality med rec is done. And that has to be separate from the strings of other data. Tricky! Great research, really like how this was presented and how easy it is to respond, looking forward to seeing the results. Many thanks.

P11: like many other areas in health IT, I believe that the use of PERM will end up taking more time (because there is more information for providers to review), but it will enable them to do a better job

P12: No further comments

P13: I think the main one missing for me is in relation to organisational culture and how this filters down across the organisation to users and how important or otherwise use of a system will be perceived. (I mentioned in my feedback to theory 9 how I think it could fit here). Overall though, I think they are very well balanced, cover the pertinent points, and generally easy to understand. Well done on what's been achieved so far! Looking forward to seeing the finished product.

P14: No comment recorded
