# Supplementary material for: Personal Electronic Records of Medications (PERMs) for medication reconciliation at care transitions: a rapid realist review
Source: BMC Med Inform Decis Mak. 2021 Nov 3;21:307. doi: 10.1186/s12911-021-01659-8 (PMC8565006; doi:10.1186/s12911-021-01659-8)
Supplement: Supplementary file 12 — Additional file 12. Full data synthesis. [file 12911_2021_1659_MOESM12_ESM.pdf]

Additional File 12: Full data synthesis

Theories

1. Engage Stakeholders 2
2. Inclusive Design 3
3. PERMs complement existing good processes 6
4. Build Trust 7
5. Tailored Training 9
8. Resource investment 13
9. Positive impact of Legislation or Governance 15
10. Patients as users of PERMs 16

1. Engage Stakeholders

Engage Stakeholders - If stakeholders including all user groups are given the opportunity to provide input, and both give and receive feedback at all stages of the design, implementation and use of PERMs, they will feel engaged, be supportive and understand the challenges, they will then accept and feel confident about using PERMs to complete MedRec at care transitions.

There was a general consensus from the studies that engagement of the stakeholders was an important element of the successful design, implementation and use of PERM for MedRec. The level of engagement was a factor, it is not enough to have everyone involved, they must be facilitated to provide input in a meaningful way, all levels of stakeholder must be engaged, bottom up and top down, workshops that allow cross department discussions so that issues are not siloed were considered helpful and it was felt important that technology and clinical elements should be considered equally.

> *The results of this study are of particular importance in the context of the implementation of a future e-Medikation system or comparable applications, as they indicate that the group of health care professionals can be quite heterogeneous and different factors for acceptance or rejection may exist in different subgroups. Therefore, it is crucial to carefully analyze project-specific requirements in general as well as to consider the specific requirements and concerns of the different subgroups within the 'health care professionals' stakeholder group. Although this seems obvious, this important step is often neglected in practice, which can significantly influence the subgroup's perception of the whole project and its goals.* Hackl et al 2014
>
> *There are two reasons, based on analysis of the data, for the lack of users' involvement; firstly, stakeholders and communications management influenced users' involvement. This effect is rooted in the NHS organisational culture and structure that restricts and confines bottom level roles to merely implement what comes from the upper managerial levels. This is because most NHS organisations have top-down hierarchical structures that restrict the free flow of information between the upper and the lower managerial levels. Secondly, clinicians' attributes prevented end users from being involved in the implementation of LORENZO.* Salloum 2011

Giving users the opportunity to give feedback at the design stage in relation to what works, what doesn't work, where potential errors could be created and the ability to offer possible solutions was seen as a positive way to engage the users and also to identify problems early. Setting up innovative ways for users to give feedback will increase their engagement in the process. Users should see their feedback being used.

> *After core functionality was developed, feedback from clinical users (including residents, attending staff physicians, and pharmacists) generated subsequent requirements. Entry into the software development queue of all new requests for*

features or functionality was prioritized accordingly, based on the impact on patient safety enhancement, improvements in process efficiency, and, finally, other clinically utility. Tamblyn 2018

Phase 1 involved "developing" a simple "EHR MedRec Issue Reporting Tool" ("Reporting Tool") to enhance the capability of an existing "SKN/Discussion Tool" (Microsoft Yammer), to facilitate knowledge exchange related to EHR MedRec and "integrating" both the Reporting Tool and the SKN/Discussion tool into the EHR workflow at AU Health. Therefore, the EHR-integrated SKN System on MedRec consists of two components: 1) Reporting Tool, and 2) SKN/Discussion Tool (Microsoft Yammer). Rangachari 2018

Approximately, 60 practitioners (physicians, nurses, and pharmacists), who have signed on to participate as "SKN Users," will report issues related to MedRec on a regular basis (over the 52-week SKN period) using the "Reporting Tool." Concurrently, a group of five "SKN Moderators" (i.e., senior administrators, including the Chief Medical Officer (CMO), Chief Medical Information Officer (CMIO), and physician champions) at AU Health will bring reported issues up for discussion via the "Discussion Tool." Correspondingly, SKN Moderators will play a key role in facilitating tacit knowledge exchange across a diverse group of practitioners, to enable engagement, learning, and practice change (EHR meaningful use). Rangachari 2018

It is important to consider the understanding of all stakeholder in relation to the use of PERM, for example the lack of clinical expertise of management and their awareness of the Med Rec processes at care transition, or the knowledge of clinical staff in relation to what is possible with an electronic system.

Because senior management people lack medical expertise and did not have enough awareness and understanding of the NPfIT [National Programme for Information Technology] overall, they were unable to provide support to the end users. Therefore, the NHS needs to have top level seniors who can support end users and champion the project towards a successful implementation. Salloum 2011

---

2. Inclusive Design

If PERMs are designed with user input and employing user-centered design & usability principles then users will feel heard and supported, thus fostering successful collaboration, acceptance and increased use of PERMs to complete MedRec at care transitions.

The types of display features identified and discussed in the studies included the number of pages and columns per page, the use of drop down options and limiting these options, the display of detailed information "on demand" to avoid clutter, whether data entry was compulsory or optional,

the methods used to aid drug identification, sorting or display and the availability and value of free text options.

Our model focused on four principles of heuristic design: Match between system and the real world; consistency and standards; flexibility and efficiency of use; and aesthetic and minimalist design. Common Internet functionality was incorporated: Use of a virtual shopping cart; and a layout reminiscent of an e-mail application. Taking action on medications in the list in several different ways was supported, to provide flexibility. For example, a user could click on an individual medication to change. Alternatively, the user could select multiple medications to change them as a group. Finally, elements for display were chosen for simplicity and aesthetic. Cadwallader 2013

A number of studies suggested the layout should be different depending on who the user was, for example patients or healthcare workers.

The patient/caregiver version had larger font, graphics to illustrate the times medications should be taken, and a section where the patient could make notes or write down questions to ask the nurse or physician. Sheehan 2018

Other issues considered included dealing with shorthand/ abbreviations in the free text and the general use of terminology in relation to drug names and use of drug databases or formularies.

Even with a limited sample from single NHS Hospital Trust, the variation of abbreviations, acronyms, and other shortened forms was significant. Other kind of shortened forms found in this study include special symbols, numeric expressions, and also metonyms. These shortened forms were found in various parts of discharge summaries, not only the medication parts. The use of special symbols is likely to disappear with the introduction of electronic data entry systems as they are more difficult to input. Kusnadi 2012

Our unique approach departs from the usual practice of sorting community and hospital drug lists alphabetically, where 2 medications that are the same or similar can appear in very different orders on the 2 lists to facilitate reconciliation and adjudication, the American Hospital Formulary Classification System was used to group drugs by pharmacologic class, and classes were then ordered by clinical importance based on expert opinion. This approach provides more clinical coherence as medications are reviewed and considered within a group with consistent therapeutic intent (eg, cardiovascular medications, anticoagulants). Drug records in the hospital and community had to be mapped to their generic molecules, then by dose per administration, frequency, and route in order for this matching and ordering to be achieved. Tamblyn 2018

If the PERM system is introduced on a phased basis the long-term benefits can be hidden from the users as not all data may be available to them.

Another issue that faced end users in terms of realising the benefits was that LORENZO's development methodology made it difficult for NHS users to see the full potential of LORENZO and to plan for future benefits. This difficulty was because the system had not been fully implemented (functional), in terms of deploying the whole set of deployment units, and thus resulted in the incapability of deployment units to communicate with each other. Salloum 2011

If complex systems are required to cater for the needs of multiple users, it is important to develop these early on in the design process otherwise users will lose confidence in the system.

Our second design consideration involved the ease of identifying medication list changes. Without discrepancies between medication sources, reconciliation is straightforward. We therefore focused on more complex, yet common, scenarios identifying five categories requiring providers' special attention. Cadwallader 2013

Analysis of the data collected presented an evidence of an IT failure in the NHS due to the failure of the system to take into serious considerations the numerous information requirements of the various user groups in NHS organisations. Ignoring end users' requirements led to workarounds and threats to system security. Thus, adopting more user-centric approach for developing computer systems enhances the success of such systems. Salloum 2011

Many studies reported the existence of a varying degree of electronic systems in use before the introduction of a new or wider system. It was unclear if this was a help or a hindrance to the process.

All hospitals and municipalities in the region had implemented EPR systems; however, the systems were not compatible between the hospital and the primary care setting. Fogged 2018

During the past decade, three quarters of all counties in Sweden have implemented shared medication lists for their health care (hospital, psychiatry, primary care), by sharing EHR system [10]. There is no automatic transfer of information on patients' medications between EHRs in different counties. Hammar 2014

The Pharm2Pharmmodel, with the above roles and responsibilities, was launched and implemented for 7 months, spanning three counties, with very basic technology, specifically the hospital EMRs (with the exception of one hospital that

was still using paper records at the time of launch), cell phones, and fax machines. Pellegrin 2018

---

3. PERMs complement existing good processes

If PERMs complement MedRec cognitive and workflow processes or forms that are already in existence in a setting and have been shown to work well, then PERMs will feel familiar and consistent, users will feel confident using them and PERMs will become embedded more easily into normal work practices, allowing a smooth transition to PERMs to improve MedRec at care transitions.

The importance of firstly ensuring that the system being used for Med Rec is fit for purpose before any plans to design a PERM system was stressed in a number of studies. Any failure of the Med Rec system will be blamed on the PERM system and created negative feelings towards it.

The move to electronic discharge summary systems was anticipated to solve the longstanding problems associated with poor data quality and reduce delay in the production and transmission of discharge summaries between secondary and primary care health care providers in the UK National Health Service. A consequence of investment in a national IT infrastructure for electronic health records has focused attention on template design and the IT system requirements. The routine practices of doctors involved in discharge summary construction, and other factors that contribute to the problems of delay and data quality, have been less well explored.

This study aimed to gain an understanding of paper-based discharge summary construction in a secondary care context in order to identify and analyse the implications for improving electronic discharge summary systems, and potentially avoid inadvertent transfer of inherent problems. Kusnadi 2012

One of the Issue that arose in the largest number of studies was that of whose responsibility it was to do MedRec. The practice of junior doctors signing off on medications for patients they had not been caring for was an issue of concern as was the delays experienced by pharmacists while waiting for the discharge summary to be completed by the doctor. The introduction of a PERM system will not solve these issues and some agreement on the process must first be achieved.

In the initial workflow model, the physician discharging the patient took full responsibility for prescribing all medications at discharge after the reconciliation process was completed. However, many physicians were concerned about renewing or modifying community medications they did not start, for which they did not know the indication, where they may have disagreed with the medication's use but did not want to stop it, or where they did not want to be the

physician on record as the last prescriber for a chronic medication that would require refills. As a result, surgeons commonly wrote "continue all previous home medications" on the paper discharge prescription. Tamblyn 2018

Several physicians stated that being responsible for a correct and accurate list was one matter, but to be responsible for that all the prescribed medications were appropriate for the patient was an entirely different matter. ''I feel like I'm responsible. But if an ophthalmologist for example has prescribed an eye drop for the pressure in the eye it's hard for me to take responsibility for that prescription. But it is my responsibility to make sure that the list is correct.'' Hammar 2014

Various findings in this study indicate that senior doctors demonstrated a low level of availability, priority and accountability to ensure that high data quality discharge summaries were produced in a timely manner. The indications include the tendency to delegate the completion of TTOs to an available junior doctor, most often without any supervision or further validation. The completion of the full discharge summaries was often seen by senior doctors as less important compared to the other commitments. Kusnadi 2012

Additionally, within the hospital context, the assignment of MedRec responsibilities among provider subgroups—multiple physicians, nurses, and pharmacists, —is often unclear, leading to inefficiency and potential for error. Rangachari 2019

---

4. Build Trust

If users are aware and understand how they and others access and use PERMs, the integrity of the PERMs data sources and the data protection controls, their trust and confidence in the system design and use will increase, they will then be more likely to value and use it at care transitions to improve MedRec and patient safety.

Distrust in PERM systems was reported as being mainly based on a lack of understanding of the processes or roles of others. But trust in the sources and accuracy of the data were also an issue (patient recall, pharmacy data; Rx V adherence).

The physicians had different views on the reliability of the medication list, from perceiving it to be reliable most of the time, to deeming it completely unreliable. Hammar 2014

Absence of shared understanding of how the EHR MedRec system is being used by other clinicians. For example, outpatient subspecialists are not convinced that MedRec marked as complete in the system at the time of hospital discharge translates to a complete and accurate medication list. Absence of shared understanding of the why, i.e., the value of EHR MedRec in preventing

discrepancies and promoting patient safety. For example, outpatient providers expressed the concern that inpatient clinicians may not realize the importance of ensuring a medication list that is free of discrepancies at discharge, to enable patients to effectively transition into the community. Rangachari 2018

However, it was revealed from an analysis of the data that the NHS did not do enough in practice to translate this feature into practical procedures such as assessing risk incorporated with the execution of clinical processes. The author thinks that assuring clinical safety needs clinical processes to be well standardised, clear and understood by the end users and the LSP [Local Service Provider]. Salloum 2011

Relying on the patient as the sole source of medication adherence information, can also present problems due to "recall bias, overestimation of adherence, and elicitation of socially acceptable responses" … clinicians make assumptions about medication-list accuracy and patients' adherence based upon electronic documentation that may itself be inaccurate. Cadwallader 2013

Yet, even aggregated pharmacy dispensing databases have limitations that preclude their acceptance as a gold standard source for active medications. First, data from these aggregated sources can be expensive. Second, medications such as over-the-counter medications may still be omitted (though this is true less often as medication data processing systems become more advanced). Finally, 16% of new prescriptions are not filled. Pharmacy data, therefore, do not perfectly correspond to active orders. Cadwallader 2013

Trust issues also related to the safety of the information and the reliability of the system and the potential for it to breakdown. It is important that these issues are included in any education or training around the introduction of a PERM system (Tailored Training Theory).

The physicians perceived that the increased availability of information resulted in an increased risk of violation of patient privacy and that a larger database of information implied an increased risk of unauthorized access of information.

There was still a risk for events limiting the availability of information, such as system breakdowns. These events were regarded as rare and not as the results of shared medication lists. Nevertheless, when occurring, system breakdowns were problematic and could pose a risk to patient safety due to insufficient back-up routines. Hammar 2014

Data transfers occur through secure connections and data themselves are encrypted before their transfer. The app can only be used for patients registered to the ReHN [Regional eHealth Network] by a HCP taking care of the patient. Marien 2019.

The author argues that system security is one of the factors that may directly affect clinicians' attitudes toward the use of LORENZO. This argument is based on the fact that end users were mostly concerned with the extent to which the clinical content is secured against unauthorised access and/or information misuse, and that patients' privacy is assured… the author perceives information privacy and confidentiality as pivotal to the design, and implementation of a HCIS [Health Care Information System] that could gain the acceptance of end users .
Salloum 2014

---

5. Tailored Training

If training is provided to users that takes into account their existing MedRec knowledge and skills, their computer skills and their role at care transitions, and the training outlines the clear benefits, usefulness and usability of PERMs, users will then feel less anxious and be more engaged and confident in relation to the introduction of PERMs in their setting.

There was a general consensus regarding the need for training on any new system, with some studies outlining what this training should look like. If users are made aware of the errors that can occur during Med Rec at care transitions and/or acknowledge that a problem exists, they will better understand the need for change and how the PERM system aims to improve safety. A supportive culture including no blame reporting within the organisation was shown in one study to foster learning and improved safety.

In effect, the results suggest that meaningful use of EHR MedRec could be facilitated by creating shared understanding of the process for MedRec and responsibilities for each step of the process among all practitioner (stakeholder) groups involved in the EHR MedRec process. Additionally, it would be important to create shared understanding among practitioner groups, of the value of EHR MedRec in preventing medication errors and discrepancies during care transitions and promoting patient safety. *Rangachari 2018*

The author believes that users' anxiety in using the system was rooted in their lack of the required informatics experience. Consequently, enhancing users' IT experience would enable them to perceive the real value and benefits of the system, and to reduce their anxiety. The author bases his argument on the grounds that, when users have sufficient level of IT skills and knowledge, they can deal more easily with the new deployment units and be more informed about their advantages. Salloum 2011

Nurses and pharmacists attended education sessions before study initiation. Nursing education was provided by investigators at staff meetings and individually. A flow chart was created to guide nurses through the admission and discharge medication reconciliation documentation process. All pharmacists attended a three-hour, hands-on computer education session. Before pharmacists were scheduled to work on the study unit, they completed an electronic

medication order-entry competency evaluation covering admission through discharge using a test patient. Posters were placed on the medical unit to educate physicians about the medication reconciliation process, including information on how to view medications in the CPCS, what to complete on the reports, and whom to contact with questions. Individual education was provided for physicians who frequently admitted patients to the unit. Kramer 2007

This study suggests that improving the competency of junior doctors is necessary if they are to be involved in the completion of effective discharge summaries. This can be achieved through formal education and training about discharge summary record keeping in combination with audit, feedback, and supervision in completing discharge summaries. Kusnadi 2012

Across these health IT tools, a recurrent barrier to adoption was the lack of experience and comfort with technology among some of the community pharmacists. Pellegrin 2018

Older users are less inclined to use IT than younger ones. This notion requires that the NHS undertake intensified training programmes for older employees who seem to be resisting the system…. Because most of the end users' skills reside in the clinical side rather than the technical side of the project, as was discussed in section 8.3 .6.1.3 of the Analysis of the Collected Data chapter (Lack of End Users' Informatics Experience), the author thinks that health informatics departments should provide training programmes and assistance to clinicians to improve their technical experience in dealing with computerised health solutions. Salloum 2011

---

6. Support and on-demand training

If support and training on PERMs is available on demand to cater for new staff or those needing additional support, at times or in formats that suit all users, with the opportunity for users to give feedback on the training, then the users will feel supported and enabled to use PERMs consistently thereby improving MedRec at care transitions.

A number of studies highlighted the need for continual or repeat training to cater for new staff, staff turnover or those needing additional support. This repeat training would also ensure that the standard of Med Rec would remain consistent.

Nearly half of respondents were not satisfied with the official information regarding e-Medikation they received at the start of the pilot project, and around one third of respondents were not satisfied with the initial user training they received. Ammenworth 2014

Provide ongoing education and training to doctors to improve the medication reconciliation process so that all changes made to eDPs are re-imported into the eDS to provide accurate information to support continuity of care. Ng 2013

The medication management training has since been translated to an online, self-guided program "Identifying and Resolving Drug Therapy Problems across the Continuum of Care" (http:// pharmacy.uhh.hawaii.edu/ce/irdtp.php). Pelligrin 2018

Although training sessions were conducted with nurses prior to the pilot, our feedback identified areas in which training could be improved including extending training to include physicians and providing more information to home healthcare nurses including specific examples of insights. The training of multiple user groups and a larger insights section have both been incorporated into training which is now provided via an e-learning module designed to teach users how the HOME tool should be used, encourage simplification of regimens, and improve patient and family understanding of medications. Sheehan 2018

There are several factors that we believe contributed to our success. Support was provided by senior hospital and clinical unit leadership, clinical champions existed at the unit level, field staff provided ongoing training and feedback to the scientific and development team about technical, usability, and professional issues, weekly adoption rates were analyzed, and the development team responded to modify the application and system to address priority issues. Tamblyn 2018

The studies also reported on the value of allowing some change in job description for key staff during development, implementation and continued use. Staff would be freed from their normal duties to become supper users and champions to support and encourage their colleagues or as monitors to ensure the correct processes were being followed during implementation and general use.

Field staff first trained the champion users on the unit, usually those with the primary responsibility for medication reconciliation, then gradually expanded training and support for primary users: residents, pharmacists, nurse practitioners, medical and pharmacy students, and pharmacy technicians, and eventually staff physicians and nurses who played a smaller role in the day-to-day process. Tamblyn 2018

Similar levels of training must be available in the use of PERM once it is developed. The opportunity for staff to provide feedback in relation to the training offered to them is also important.

7. Interoperability

If the data sources are technically interoperable with PERMS, allowing integration of data from multiple sources then users will find PERMs align with or improves the MedRec process flow, thereby increasing their use of PERMs for MedRec at care transitions impacting positively on patient safety.

Interoperability was assumed in many of the studies, will little reference to it other than to define it and to acknowledge that the ability to integrate data from multiple sources was important in the usability of any PERM system.

In this national level implementation, electronic discharge reports have to be specified according to strictly defined interoperability standards to ensure the records can be used safely across different care settings and clinical applications.

The second significant change is the emerging paradigm of "connect all" as an alternative to the unsuccessful NPfIT "replace all" approach. While the "replace all" approach attempts to "force" NHS Hospital Trusts to replace their IT systems, the "connect all" approach focuses on facilitating the connectivity between existing IT systems used by the NHS Hospital Trusts. This new directive spurred the development of the NHS interoperability toolkit (ITK), which provides interface specifications for linking the different pieces of existing software systems. Kusnadi 2012

The benefits of using a PERM system to improve the safety and efficiency of MedRec at care transitions needs to be apparent to the users.

The other unique aspect of e-Medikation is the integration of the national medication information into the local EMR systems: Physicians' EMR systems automatically download e-Medikation information and then update the local medication history of a patient based on this information. The complete medication history (at least the information stored in e-Medikation) is thus integrated in the local EMR, and all interaction and inventory checks can be performed on the complete medication history of a patient. Ammenworth 2014.

Most physicians believed a nationally shared medication list would further increase patients' safety, if the technical solution and implementation were carried out correctly and smoothly.

''It depends on how it is linked to our system. If it is something new where I have to add information, I don't think it will work. Because it will be another system you have to work with. But if there is an automatic link it might work.'' Hammar 2014

Interoperability: To be viable, the solution must be able to fit within current workflow situations. Although difficult to imagine within our current EHR

landscape, effort is currently underway towards interoperability. We must be forward thinking and design for such a future. By using the same infrastructure an EHR uses to pull information from a Surescripts® Medication History data source, the EHR would also be able to pull information from the medication manager. In addition, as an EHR is able to send an e-prescription to a pharmacy, it would also be able to send a message to the medication manager. The medication manager would have the capability to consume HL7-NCPDP SCRIPT e-prescriptions, the National Council for Prescription Drug Programs standard for transmission of prescription information22-27 to update information. In the future, it could also run as a SMART application28 utilized as a web-app, mobile device-app or trusted service within an EHR. Pandolfe 2016

The PERM system must be seen to support the existing MedRec processes.

Improvements concerning workflow integration, usefulness and interoperability clearly appeared to be crucial to achieve users' acceptability. Marien 2019

---

8. Resource investment

If the increased effort, volume and quality of data gathered when using PERMs for MedRec at care transitions, providing opportunities for risk identification, management and analysis, is recognised by leaders/ management from the outset then they will understand the need for additional resourcing to support the use of PERMs to improve MedRec at care transitions and patient safety.

Improvement does not come without a price, although improving the safety of the processes MedRec the introduction of a PERM system also increases the workload. A number of studies reported on the need for additional staff or increased budgets to pay for additional staff.

Pharmacists spent a median of 5 (range 2–16) minutes per patient contributing to EDSs. Logistics, timing and pharmacist workload were barriers to delivering the intervention. Additional staff resources is needed to enable pharmacists to consistently deliver this effective intervention. Elliott 2020

a centralized system aids the institutions and individuals attempting to reconcile medications at every transition of care. The process of reconciliation requires significant time and resources. It is a process that is repeated often and at every outpatient visit or transition of care. Pandolfe 2016

The principal way in which Isoft had affected this aspect of their work was to make it more time consuming. This is in keeping with the findings of Murray et al, that following implementation of a system for computerised outpatient prescribing, the time pharmacists spent checking prescriptions entered by

physicians and dealing with problems increased when compared with the paper system. Smith 2018

To facilitate interoperability the need for agreement and co-ordination in relation to drug names and the use of drug databases or formularies has already been addressed under the theories "Collaborative Design" and "Interoperability". However, it worth mentioning here that resources would be required for these processes.

A number of studies highlighted the point that the existence of a PERM system alone was not enough to improve the safety of Med Rec at care transitions. Dialogue between patient and HCW and between HCW and HCW is still considered a vital component of the process. In addition, as mentioned earlier the PERM will only be as good as the Med Rec system it supports.

To understand which medications they actually were taking, the physicians regarded it as important to have a good dialogue with patients regarding their current prescriptions; their dialogue with the patient had not changed with a shared medication list. Hammar 2014

Implementing shared systems for health information has turned out to be more challenging, complex and expensive than expected. The major issues are usually not related to technology, but rather strategies and management. Hammar 2014.

Managing expectation was also a topic discussed, one such topic was the expectation that introducing a PERM system would eliminate the use of paper files. This in fact was found not to be the case, many PERM systems were still shown to rely on paper at different stages of the process, most commonly in the provision of a discharge summary to a patient.

---

while all prescriptions were entered in the health care provider's EMR and communicated to e-Medikation, the patient still received a print-out of the prescription and took this paper-based prescription to a pharmacy. Ammenworth 2014

Inpatient medication administration records were electronic (paperless), while discharge prescriptions were prepared electronically then printed on paper. Elliott 2020

One striking observation from this case study was the amount of paper produced from this electronic system. Dispensary processes and the work of the pharmacists on the wards relied upon documents printed from PCIS. Smith 2018

The finalized discharge prescription is printed, signed, and given to the patient, and a copy is scanned into the hospital chart. A letter summarizing the changes made to the patient's community drug list is faxed to each of the community-

based dispensing pharmacies and prescribing physicians identified from the dispensing data. Tamblyn 2018

---

9. Positive impact of Legislation or Governance

If the introduction of PERMs or standards for the MedRec process are supported by relevant legislation, governance or policies then organisational participation and engagement is increased impacting positively on individual users' engagement with the introduction of PERMs to improve MedRec at care transitions.

Existing legislation or governance was mostly seen as a positive influence, however if the legislation or governance was not adapting to changes in the expanding role of healthcare workers it could have a negative influence. In addition, the importance of having professional bodies such as medical, nursing or pharmacist associations engaged in the process was reported in one study, if they are not supportive of the initiative it can be a major challenge.

A European survey showed that critical success factors for national eHealth projects comprise four categories: first, the political dimension, including a national eHealth strategy, clear legislation, and sufficient funding: Ammenworth 2014

In line with the NPfIT plan to reform NHS IT systems, NHS Hospital Trusts in England are now obliged to deliver discharge summaries to GPs within 24 hours after a patient discharge (Department of Health, 2008). The policy essentially requires NHS Hospital Trusts to implement an electronic discharge summary system with connectivity to the GP system. Kusnadi 2012

Health information technology (IT) in support of medication management has focused primarily on physician use. This is not surprising given that physicians have been a primary target of federal incentives to adopt electronic health records and that generating and transmitting prescriptions electronically has been a key Meaningful Use objective under the CMS Electronic Health Record Incentive Program. Pellgrin 2018

In preventing technician involvement in the discharge process, Medisec hindered the very developments in pharmacy working practices that recent Government policy has sought to encourage. Smith 2016

By 2017, the hospital was expected to have implemented medication reconciliation at admission, discharge, and transfer to receive accreditation. Tamblyn 2018

---

10. Patients as users of PERMs

If patients are supported to use PERMs to understand and record their medication use and share their medication information, they will feel enabled, empowered and organised in helping to maintain an accurate medication record, be more informed and have improved likelihood of adherence to their medications.

There were mixed feelings in relation to the role patients could play in Med Rec. A number of studies reported the successful use of a patient completed version of PERM which increased their understanding of their medical conditions and the purpose of the medications they had been prescribed and improved their adherence to them.

Patients who had their medications electronically reconciled reported a greater understanding of the medications they were to take after discharge from the hospital, including medication administration instructions and potential adverse effects. Krammer 2017

Most importantly, by allowing the patient access to the same list used by providers, patient input into the list will more accurately reflect the daily medications actually taken and allow safer prescribing practices.

A well-designed medication manager can provide services such as brand and generic name, dose, frequency, images of the medications, common indications, convenient links for more detailed drug information, and an interaction checker. The combination of these services will aid in patient-education in addition to recognizing possible unintentional polypharmacy. In addition, the medication manager will provide an opportunity for dialogue regarding differences in the way that medications are prescribed and taken.

Patients are willing and able to interact with electronic medication tools to manage their health. Pandolfe 2016

The concerns that were highlighted related to the potential for error if the patient's recall was inaccurate or they provided acceptable responses rather than truthful responses. One study showed that patients over 80 years were less likely to engage with a PERM and some older adults express fear of using a PERM in case they inadvertently deleted data entered by others.

Relying on the patient as the sole source of medication adherence information, can also present problems due to "recall bias, overestimation of adherence, and elicitation of socially acceptable responses" Cadwallader 2013

While the CCT was well accepted by those in their 60s and 70s, the oldest adults in our study (80+ years old) declined to use any electronic medication management system, regardless of how simplified its interface might be.

For instance, users found it confusing to view their own personal medication list and the medication lists from various doctors. Comparing their list and a doctor's list side-by-side was more difficult than helpful – participants were concerned that they were inadvertently corrupting their doctor's medication list. Seik 2010

In relation to the patients providing consent to other to access their medical records, many were not aware that healthcare workers needed this consent, most were happy to give it and felt reassured that the information was available to those who cared for them. Some reasons given by patients for not giving consent were patients who did not wanted to disclose some medications or details of their medical history to others.

The majority of the physicians had experienced few or no patients who had ever had concerns with a physician being able to access their medication information; quite the opposite, patients were usually surprised to hear that the physician had not always had access to the information. Hammar 2014

However, some patients do not want to share information concerning their mental health, sexual health and gynecological problems in a shared health record system. Hammar 2014

Twenty-six percent of physicians and 14% of pharmacists reported that "from time-to-time" or "frequently" patients used the opportunity to exclude drugs from being recorded within e-Medikation. Ammenwerth 2014
